# Association of long-term care facility ownership and location on residents’ mortality and hospitalisations: A population-based retrospective cohort study in Australia

**DOI:** 10.1136/bmjopen-2026-117917

**Published:** 2026-09-20

**Authors:** Miia Rahja, Ling W Davies, Robert N Jorissen, Maria Crotty, Craig Whitehead, Keith Evans, Megan Corlis, Carly Meyer, Gillian E Caughey, Maria C Inacio

**Affiliations:** 1 College of Medicine and Public Health, Flinders University, Adelaide, South Australia, Australia; 2 South Australian Health and Medical Research Institute Limited, Adelaide, South Australia, Australia; 3 Rehabilitation, Aged and Extended Care, Flinders Medical Centre, Bedford Park, South Australia, Australia; 4 Australian Nursing and Midwifery Education Centre, Ridleyton, South Australia, Australia; 5 Bolton Clarke, Kelvin Grove, Queensland, Australia; 6 College of Nursing and Health Sciences, Flinders University, Adelaide, South Australia, Australia

**Keywords:** Nursing Homes, Mortality, Health Services for the Aged, Hospitalization

## Abstract

**Abstract:**

**Objectives:**

Long-term care facility (LTCF) ownership and location can influence resident outcomes in multiple ways, with concern that they are associated with quality of care and resident outcomes. The aim of this study was to assess whether LTCF ownership was associated with risks of mortality, emergency department (ED) presentations, unplanned hospitalisations and hospital days during the first 12 months after LTCF entry, and whether these associations varied by geographical region in Australia.

**Design:**

A retrospective propensity score-matched cohort study.

**Setting:**

Registry of Senior Australians National Historical Cohort (2013–2018).

**Participants:**

Non-indigenous LTCF residents aged ≥65 years.

**Exposure:**

LTCF ownership type: government, not-for-profit or for-profit. Analyses were stratified by LTCF geographical remoteness: metropolitan, inner regional or outer regional/remote/very remote area.

**Primary and secondary outcome measures:**

Cox proportional hazards, Fine-Gray competing risks and negative binomial models were employed. We report adjusted HRs, sub-distribution HRs (sHR), rate ratios, CIs and cumulative incidence estimates.

**Results:**

Of 205 079 residents studied, 8961 (4.4%) lived in government-run, 105 144 (51.3%) in not-for-profit and 90 974 (44.4%) in for-profit LTCFs. Compared with residents in not-for-profit LTCFs, mortality risk was higher in for-profit (HR=1.17, 95% CI 1.09 to 1.26) and government facilities (HR=1.20, 95% CI 1.08 to 1.35) located in inner regional areas (24.1% of residents). Residents in government LTCFs had lower ED presentation risk than residents in not-for-profit (sHR=0.68, 95% CI 0.57 to 0.82) and for-profit (sHR=0.61, 95% CI 0.52 to 0.72) LTCFs. Similar observations were made for unplanned hospitalisations and hospital days, and across regions.

**Conclusions:**

Government LTCF residents had fewer hospital-related events, while not-for-profit residents had lower mortality, with differences most evident in inner regional areas. Ongoing monitoring of LTCF ownership trends and their association with resident outcomes is vital for policy development.

**STRENGTHS AND LIMITATIONS OF THIS STUDY**

- The use of population-based linked registry data minimised loss to follow-up and supported comprehensive capture of outcomes.
- Propensity score matching was used to balance resident- and facility-level characteristics across ownership groups.
- Residual confounding may remain due to unmeasured variables.
- Propensity score matching reduced the analytic sample to residents with comparable characteristics.

## Introduction

 Long-term care facilities (LTCFs; nursing or residential aged care homes) offer personal, medical and 24-hour nursing care for older individuals who can no longer live safely at home.^1^ Globally in 2021, there were approximately 42 LTCF beds per 1000 individuals aged 65 years and older.^2^ Although LTCF use has declined over the past decade, the total number of individuals accessing these services is projected to grow over the next 15 years, driven by the ageing population.^2 3^

The ownership structures of LTCFs vary across countries. In the USA and England, for-profit organisations operate over half of the LTCFs.^4–6^ In Nordic countries, like Sweden and Denmark, government municipalities have traditionally managed LTCFs, but legislative changes enabling choice models and outsourcing have led to a rise in privately owned, for-profit facilities.^7 8^ In Australia, Canada, the Netherlands and many other European countries the ownership model is mixed with a substantial not-for-profit sector.^9 10^ In Australia, over half (57.9%) of LTCFs are operated by not-for-profit providers, caring for a similar proportion of residents (57.6%) followed by for-profit (34.6%) and government providers (8.0%).^11^ Recent years have seen several mergers and acquisitions, particularly from large providers seeking to improve their sector influence, which has reduced the number of LTCF providers (from 663 in 2023 to 642 in 2024).^12^

LTCF ownership has been associated with care quality and resident outcomes, including emergency department (ED) presentations, unplanned hospitalisations and mortality,^4–6 13 14^ with not-for-profit versus for-profit comparisons favouring not-for-profit LTCFs with lower mortality and hospitalisation rates.^4 6 14^ During COVID-19, for-profit LTCFs had higher rates of infections and deaths.^15^ In Australia, a federal review into the quality of LTCFs descriptively examined 51 quality and safety indicators and found that government LTCFs had better resident outcomes on 31 indicators, including ED presentations, unplanned hospitalisations, falls and fractures, medication-related events and premature death compared with not-for-profit and for-profit providers.^16 17^ While geographical differences were also observed, ownership-based trends were more consistently reported.^17^

While care quality within LTCFs has been described in Australia,^18 19^ no comparative investigation has examined whether LTCF ownership is associated with resident outcomes using population-based datasets and sophisticated confounding adjustment methods. Such analysis could provide valuable insights into how ownership types influence resident outcomes, contributing to the development of improved care strategies and policies. The aim of this comparative study was to assess whether LTCF ownership type and geographical remoteness (metropolitan; inner regional (ie, non-metropolitan area with reasonable access to services and infrastructure); or outer regional/remote/very remote area) were associated with resident outcomes, specifically risk of mortality, ED presentations, unplanned hospitalisations and days in hospital in the first 12 months following LTCF entry.

## Methods

### Study design, setting and data sources

A retrospective, propensity score-matched cohort study was conducted using the Registry of Senior Australians (ROSA) National Historical Cohort (2013–2018),^20 21^ a platform containing de‐identified linked information about older individuals assessed as eligible for government-subsidised episodic and long-term home-based care (known as aged care) and care in LTCFs. Data collections in ROSA used included: Australian Institute of Health and Welfare’s (AIHW) National Aged Care Data Clearinghouse (NACDC), Australian Government’s Medicare Benefits Schedule (government-subsidised healthcare services) and Pharmaceutical Benefit Scheme (government-subsidised medicines dispensed), and three states (New South Wales (NSW), Victoria (VIC) and Queensland (QLD)) health authorities’ ED and inpatient hospitalisation records. Data collection from the AIHW NACDC included aged care eligibility assessments, care needs assessments at entry into LTCFs, episodes of aged care services and National Death Index (NDI).

### Study cohort

All non-Indigenous (not identifying as Aboriginal or Torres Strait Islander) individuals aged 65 and above who had a first-time entry as a permanent LTCF resident in NSW, VIC and QLD (capturing 77.7% of LTCF services in Australia)^11^ were included. Individuals with Australian Government Department of Veterans’ Affairs (DVA) entitlements were excluded as they receive subsidised healthcare services differently. Residents requiring palliative care (n=11 049) were also excluded.

### Exposure of interest

The exposure of interest was LTCF ownership categorised into not-for-profit, for-profit and government-operated facilities according to the organisation type reported.^11^

All residents were stratified by LTCF area remoteness: metropolitan; inner regional; or outer regional/remote/very remote area using the Australian Bureau of Statistics Remoteness Areas.^22^

### Outcomes of interest

The outcomes of interest were mortality, ED presentations, unplanned hospitalisations and number of days in hospital due to unplanned hospitalisations in the first 12 months after LTCF entry. All-cause mortality was ascertained using data from the NDI. ED presentations and unplanned hospitalisations were identified from the state health authorities’ records. Unplanned hospitalisations were identified from the hospital records where the ‘admission urgency status’ was indicated as ‘emergency’, not ‘scheduled’. The number of days spent in hospital was calculated as the total days spent in hospital during unplanned admissions.

### Covariates

Potential confounders, ascertained at LTCF entry, included LTCF location (state), year of care entry, sex, age, partner status, use of respite care immediately prior to LTCF entry, Socio-Economic Indexes for Areas relative socioeconomic disadvantage index,^23^ history of health conditions (dementia, delirium, malnutrition, incontinence, pressure injuries and depression identified from LTCF care needs assessments) and comorbidity burden (number of health conditions estimated using pharmaceutical-based comorbidity index Rx-Risk-V).^24^ The number of medications dispensed identified using the *Anatomical, Therapeutic and Chemical classification* codes (fifth level chemical substance)^25^ and exposure to high sedative load (ie, cumulative score of medications with sedative properties ≥3),^26^ in the 90-day period prior to the LTCF entry were also examined, and the number of hospitalisations (any, unplanned and potentially preventable) and ED presentations in the 12 months prior to LTCF entry. Primary healthcare attendances (general, after hours and urgent after hours), health assessments, management plans or pain and geriatric medicine attendances in the 12 months prior to care entry were also examined (see online supplemental table S1 for coding).

### Statistical analysis

Within each geographical group, propensity score matching was used to create comparable groups of residents to allow for the comparison of for-profit versus not-for-profit, government versus not-for-profit and government versus for-profit ownership models’ association with the outcomes of interest. Propensity scores, representing the probability of accessing each type of LTCF (not-for-profit, for-profit, government) accounting for observed baseline characteristics, were estimated using a logistic regression model. Due to a small amount of missing data (<0.1%), a complete case analysis was conducted. Subset selections were then performed using greedy nearest neighbour matching without replacement, with a 0.05 calliper to ensure that only residents with the most similar baseline characteristics were matched. The balance of the covariates before and after matching for each ownership comparison was assessed using common support plots, Love plots and average standardised mean differences (ASMD), using <0.10 as the threshold.

A Cox proportional hazard regression was employed to estimate the risk of mortality within 12 months of LTCF entry, with HRs and 95% CIs reported and cumulative incidence functions plotted. Cluster effect of LTCF was included to estimate the robust variance, assuming the presence of correlations among observations within each LTCF. Fine-Gray models were employed to estimate cumulative incidence of ED presentations and unplanned hospitalisation, with death treated as a competing event. Sub-distribution HRs (sHR) and 95% CIs were reported, and cumulative incidence functions were plotted. Proportional hazard assumptions for all models were examined using a proportional hazard test and were met and no time-dependent effects are reported. A negative binomial with an offset term for total follow-up time was used to estimate the total unplanned hospital days within the first 12 months of LTCF entry, addressing the potential risk of overdispersion. All analyses were performed using R V.4.3.2.

### Patient and public involvement

The Consumer and Community Advisory Committee at the ROSA Research Centre was consulted during project planning on the relevance and acceptability of the proposed study methods. Their perspectives and advice ensured the project reflects community priorities and supports outcomes meaningful to older Australians.

## Results

Of the 205 079 residents (in 2226 LTCFs) studied, 8961 (4.4%) lived in government, 105 144 (51.3%) in not-for-profit and 90 974 (44.4%) in for-profit LTCFs. A total of 141 509 (69.0%) residents were in metropolitan, 49 402 (24.1%) in inner regional and 14 168 (6.9%) in outer regional/remote/very remote areas. The median age at LTCF entry was 84.6 years (IQR 79.2–88.4), 60.6% (n=124 299) were female and 51.4% (n=105 441) had dementia (online supplemental table S2). After matching, the three cohorts were comparable, with ASMD<0.10 for all variables (online supplemental tables S3-S11). The matched cohorts’ descriptive outcomes within the first 12 months of LTCF entry, including the median follow-up time, are presented in online supplemental table S12.

### For-profit versus not-for-profit comparisons

**Figure 1:**
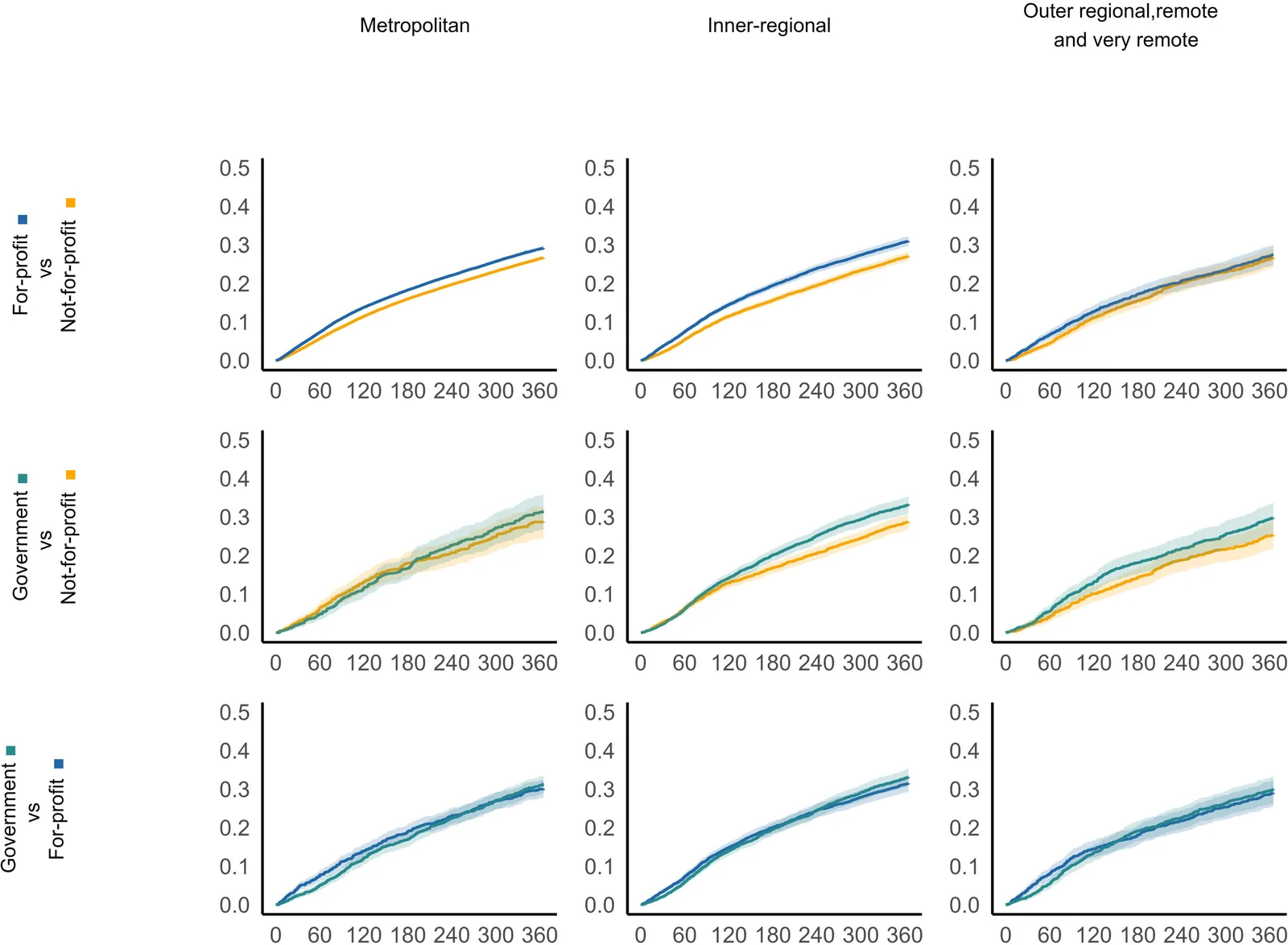
Cumulative incidence for risk of mortality. Age, sex and comorbidity adjusted cumulative incidence for risk of mortality by long-term care facility ownership type (for-profit (blue) vs not-for-profit (yellow), government (green) vs not-for-profit (yellow) and government (green) vs for-profit (blue)) and area remoteness (metropolitan, inner regional and outer regional/remote/very remote areas).

**Figure 2:**
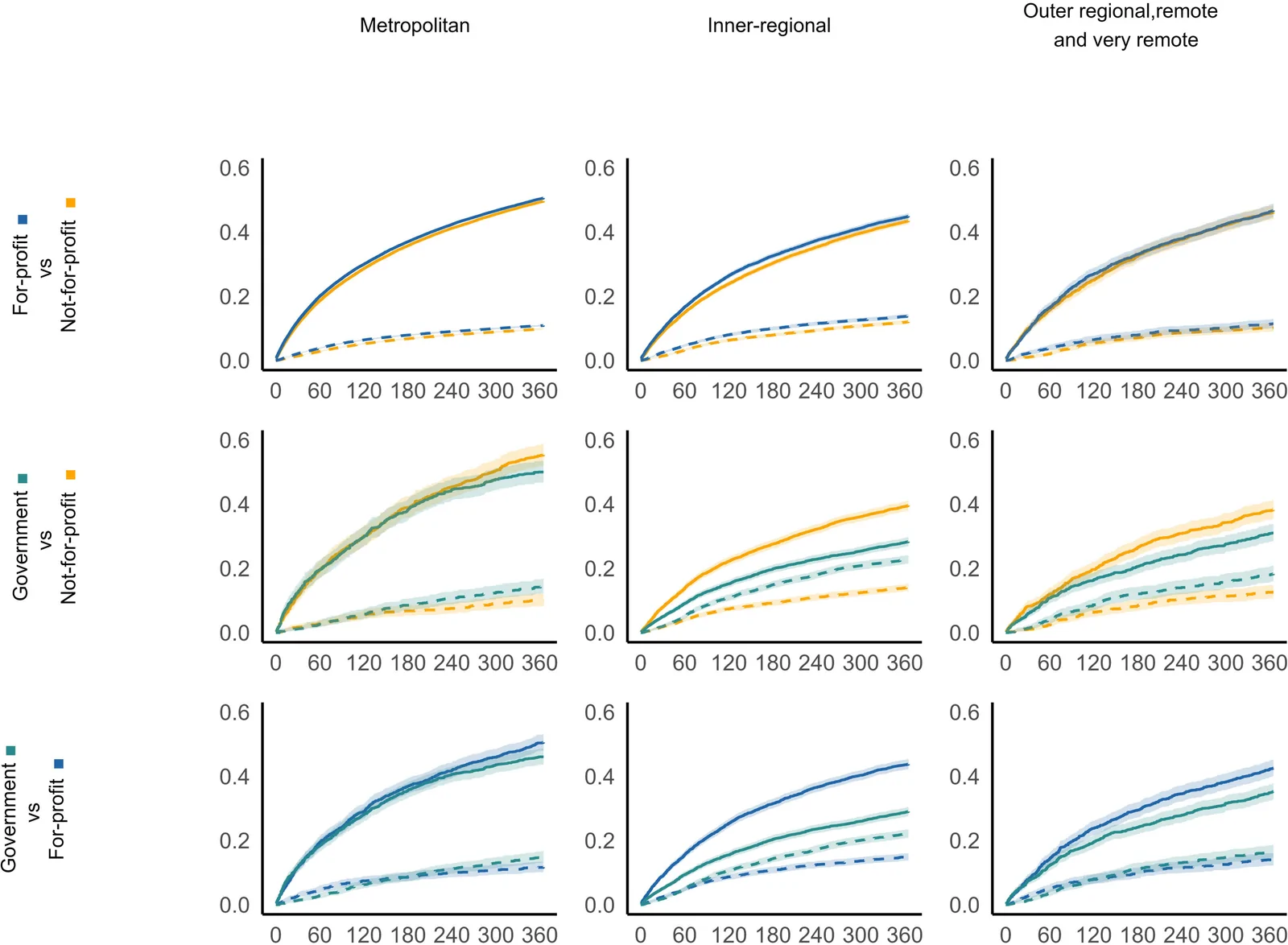
Cumulative incidence for risk of emergency department presentations. Age, sex and comorbidity adjusted cumulative incidence for risk of emergency department presentations by long-term care facility ownership type (for-profit (blue) vs not-for-profit (yellow), government (green) vs not-for-profit (yellow) and government (green) vs for-profit (blue)) and area remoteness (metropolitan, inner regional and outer regional/remote/very remote areas), accounting for the competing risk of death (dotted lines).

**Figure 3:**
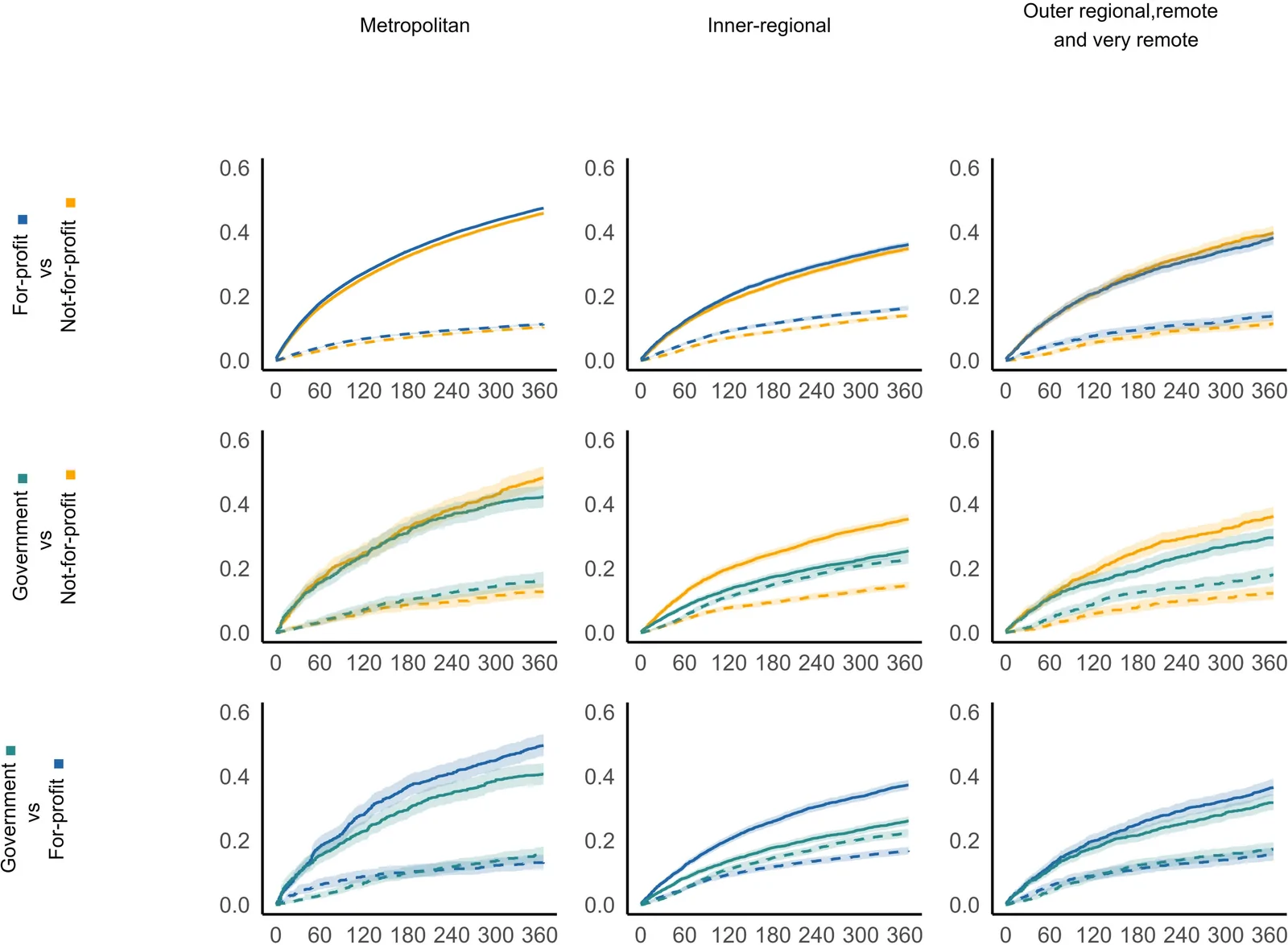
Cumulative incidence for risk of unplanned hospitalisations. Age, sex and comorbidity adjusted cumulative incidence for unplanned hospitalisations by long-term care facility ownership type (for-profit (blue) vs not-for-profit (yellow), government (green) vs not-for-profit (yellow) and government (green) vs for-profit (blue)) and area remoteness (metropolitan, inner regional and outer regional/remote/very remote areas), accounting for the competing risk of death (dotted lines).

Residents of for-profit LTCFs in metropolitan and inner regional areas had a higher mortality risk than residents of not-for-profit LTCFs (HR=1.13, 95% CI 1.09 to 1.18 and HR=1.17, 95% CI 1.09 to 1.26, respectively; figure 1, table 1). There were no statistically significant differences in the risk of ED presentations (figure 2) or unplanned hospitalisations (figure 3). If hospitalised, residents of for-profit LTCFs in metropolitan areas spent more days in the hospital compared with residents of not-for-profit LTCFs (RR=1.04, 95% CI 1.01 to 1.07) (table 1).

**Table 1:** Adjusted risks of mortality, emergency department presentations, unplanned hospitalisations and subsequent days in hospital in the first 12 months of long-term care facility entry, by area remoteness

	Metropolitan		Inner regional		Outer regional, remote and very remote	
**Mortality**	**HR**	**95% CI**	**HR**	**95% CI**	**HR**	**95% CI**
For-profit versus not-for-profit	1.13	(1.09 to 1.18)	1.17	(1.09 to 1.26)	0.99	(0.84 to 1.17)
Government versus not-for-profit	1.11	(0.89 to 1.38)	1.20	(1.08 to 1.35)	1.14	(0.96 to 1.35)
Government versus for-profit	1.02	(0.83 to 1.27)	1.06	(0.95 to 1.19)	1.04	(0.88 to 1.23)
**ED presentations**	**sHR**	**95% CI**	**sHR**	**95% CI**	**sHR**	**95% CI**
For-profit versus not-for-profit	1.01	(0.97 to 1.06)	1.05	(0.95 to 1.15)	1.05	(0.87 to 1.27)
Government versus not-for-profit	0.91	(0.77 to 1.08)	0.68	(0.57 to 0.82)	0.85	(0.62 to 1.15)
Government versus for-profit	0.90	(0.76 to 1.05)	0.61	(0.52 to 0.72)	0.80	(0.58 to 1.11)
**Unplanned hospitalisations**	**sHR**	**95% CI**	**sHR**	**95% CI**	**sHR**	**95% CI**
For-profit versus not-for-profit	1.03	(0.99 to 1.07)	1.03	(0.94 to 1.12)	1.00	(0.82 to 1.22)
Government versus not-for-profit	0.82	(0.71 to 0.94)	0.68	(0.61 to 0.77)	0.82	(0.66 to 1.03)
Government versus for-profit	0.79	(0.69 to 0.90)	0.65	(0.58 to 0.73)	0.85	(0.65 to 1.11)
**Days in hospital** *	**RR**	**95% CI**	**RR**	**95% CI**	**RR**	**95% CI**
For-profit versus not-for-profit	1.04	(1.01 to 1.07)	1.01	(0.96 to 1.07)	0.94	(0.84 to 1.05)
Government versus not-for-profit	1.02	(0.87 to 1.20)	0.83	(0.75 to 0.91)	0.92	(0.78 to 1.04)
Government versus for-profit	0.95	(0.81 to 1.11)	0.94	(0.86 to 1.04)	0.97	(0.83 to 1.13)

* If any unplanned hospitalisations.

ED, emergency department; HR, hazard ratio; RR, rate ratio; sHR, sub-distribution HR.

### Government versus not-for-profit comparisons

Residents of government LTCFs in inner regional areas had a higher mortality risk than residents of not-for-profit LTCFs (HR=1.20, 95% CI 1.08 to 1.35; table 1). Residents of government-run LTCFs in inner regional areas had a lower risk of ED presentation than residents of not-for-profit LTCFs (sHR=0.68, 95% CI 0.57 to 0.82). Compared with not-for-profit LTCFs, residents of government-operated facilities had a lower risk of unplanned hospitalisation in metropolitan and inner regional areas (sHR=0.82, 95% CI 0.71 to 0.94 and sHR=0.68, 95% CI 0.61 to 0.77, respectively). In inner regional areas, residents of government LTCFs had fewer unplanned days in hospital compared with residents of not-for-profit LTCFs (RR=0.83, 95% CI 0.75 to 0.91; table 1).

### Government versus for-profit comparisons

No differences in mortality were observed. Residents of government LTCFs in inner regional areas had a lower risk of ED presentation than residents of for-profit facilities (sHR=0.61, 95% CI 0.52 to 0.72). Residents of government LTCFs had a lower risk of unplanned hospitalisation compared with residents of for-profit LTCFs in metropolitan and inner regional areas (sHR=0.79, 95% CI 0.69 to 0.90 and sHR=0.65, 95% CI 0.58 to 0.73, respectively; table 1).

## Discussion

This population-based cohort study identified that residents of government LTCFs had a lower risk of ED presentations, unplanned hospitalisations and days in hospital compared with residents of not-for-profit and for-profit LTCFs, with minimal differences between not-for-profit and for-profit LTCFs. Residents of for-profit LTCFs in metropolitan and inner regional areas had a higher risk of mortality compared with those of not-for-profit LTCFs, and there was a higher risk of mortality in government compared with not-for-profit LTCFs in inner regional areas. While most of the differences were observed in inner regional areas, which represented about a quarter (24.1%) of the cohort studied, some were also consistent in metropolitan areas (69% of the cohort studied), and the direction of these differences was generally consistent.

A 32%–39% lower risk of ED presentation in government LTCFs in inner regional areas and an 18%–35% lower risk of unplanned hospitalisations in government LTCFs in metropolitan and inner regional areas was observed compared with for-profit and not-for-profit LTCFs, without major differences between for-profit and not-for-profit LTCFs. This lower risk of hospitalisations in government LTCFs agrees with previous Australian and Canadian research.^18 27^ An Australian study of 2900 LTCFs examining variation in care and costs found that residents of government LTCFs had less ED presentations, dementia-related hospitalisations, falls and pressure injuries.^18^ In Canada, residents of for-profit (34%) and not-for-profit (37%) LTCFs had a higher risk of hospitalisation compared with publicly owned and operated LTCFs, even after adjusting for staffing, facility size, urban location, resident demographics and case mix.^27^ However, it is likely that country-specific models for long-term care delivery, policies and geographical differences also play a role.^4 13 14^ For example, the lack of major differences between for-profit and not-for-profit LTCFs (compared with government facilities) in our study could be related to the commonalities in regulatory standards governing the provision of care,^28^ quality monitoring programme,^19^ public reporting (ie, Star Rating), models of care and workforce governance. Research on the publicly reported Star Rating system has shown that for-profit and not-for-profit LTCFs had lower odds of receiving 4- or 5-star ratings (reflecting residents’ experience, compliance, staffing and quality measures) than government LTCFs,^29^ suggesting these providers may share similarities in quality outcomes. The similarities between for-profit and not-for-profit LTCFs, particularly in contrast to government LTCFs, raise the need for close monitoring of privatisation and ownership changes to ensure resident outcomes are not compromised.^30 31^ For example, a recent study found that LTCFs acquired by for-profit providers had poorer care quality in the 2 years post-acquisition, with more hospitalisations and complaints, with outcomes especially evident in LTCFs purchased by smaller for-profit providers or those with a history of lower quality across their other LTCFs.^32^

A 13%–17% higher risk of mortality was observed in for-profit LTCFs in metropolitan and inner regional areas, and a 20% higher risk in government LTCFs in inner regional areas compared with not-for-profit LTCFs. These findings agree with previous studies reporting lower mortality rates for residents of not-for-profit LTCFs.^4 14 18^ For example, in a recent Canadian study, residents of for-profit LTCFs had a 10%–20% higher risk of mortality compared with not-for-profit LTCFs.^14^ While an earlier Australian study did not find a significant difference in premature mortality between LTCF ownership types,^18^ this is likely attributable to differences in outcome definitions, with our study reporting overall mortality. Systematic reviews, meta-analyses and cross-country comparisons increasingly suggest that not-for-profit LTCFs deliver superior care compared with for-profit LTCFs, which may explain their better mortality outcomes.^4 30^ One contributing factor could be regulatory compliance. Not-for-profit LTCFs tend to have fewer deficiencies in governmental assessments and are more likely to meet or exceed staffing and care standards.^6 33^ This adherence to regulations ensures more consistent care practices, which can be associated with mortality rates. For example, a systematic review and meta-analysis found that not-for-profit LTCFs had significantly better outcomes related to staffing.^4^ Higher staffing levels, which we did not account for, can support improved monitoring, quicker response to medical needs and better overall care, all contributing to reduced mortality.^33^ However, this does not explain why not-for-profit LTCFs performed better than government LTFCs in our study, as we earlier discussed that government LTCFs tend to perform better in Australia.^29^ Government LTCFs often serve as a safety net for individuals with more complex health needs, including those with multiple comorbidities or advanced dementia, as they accept residents who may be declined by other providers. A plausible explanation for the higher mortality rates is that these residents are already at elevated risk due to their clinical profiles. A study on the distribution of LTCFs in Australia found that government LTCFs are disproportionately located in areas of higher socioeconomic disadvantage, which may compound health risks due to environmental and systemic factors.^34^ This suggests that government LTCFs likely admit more residents from lower socioeconomic backgrounds, a group consistently associated with poorer access to healthcare, higher rates of chronic disease and worse health outcomes overall.^35^ Consequently, these residents are not only more vulnerable on admission but also more likely to die, reflecting their initial health status and the broader social determinants of health.

### Strengths and limitations

Our study is representative of the national population and likely generalisable to countries with similar LTCF (eg, New Zealand, Canada, the UK) and resident characteristics.^9 10^ We leveraged population-based linked datasets, increasing the internal validity of our analysis (through cross-checking within datasets) and ensuring limited loss to follow-up. We examined the potential effect modification experienced by the geographical location of the LTCFs, a critical evaluation in a geographically diverse and heavily urbanised country like Australia, which struggles to address care demand in less populated areas,^36–38^ and where health disparities for older people in regional/rural/remote areas are well documented.^39 40^ We also employed sophisticated analytical approaches to minimise the risk of confounding when examining the relationship between LTCF ownership type and resident outcomes.

Limitations include residual confounding, which may persist despite our approach. For example, staffing levels and resident preferences were not captured. While we included multiple outcomes (mortality, ED presentations, unplanned hospitalisations and hospital length of stay), these measures may not fully capture differences in care quality across LTCFs. Other indicators such as potentially avoidable hospitalisations or place of death were not examined. Furthermore, while we attempted to capture individuals’ care needs through the inclusion of characteristics such as detailed history of service hospitalisation prior to entering care, we did not consider residents’ level of care needs at the time of care entry. This was due to the important limitations with the available national data sources that captured residents’ care level needs in Australia during this study period. Specifically, during our study period, in Australia, care needs in LTCFs were assessed using the Aged Care Funding Instrument (ACFI). Government-commissioned evaluations, including the Alternative Aged Care Assessment, Classification System and Funding Models Final Report,^41^ identified limitations of the ACFI approach and the need for substantial redesign of assessment and classification methods for funding and care need measurement. Prior work has also highlighted variability in how ACFI assessments were applied across providers- particularly related to ownership type- raising concerns about systematic differences in reporting by facility characteristics.^42^ Given these constraints, we were concerned that the inclusion of these measures may introduce bias rather than improve comparability between LTCFs. For these reasons, and in the absence of an unbiased, nationally consistent measure of residents’ care needs suitable for this purpose, we did not include these variables in the primary analyses. We also did not consider the separate policies and regulatory requirements of the Australian states. For example, Victoria has the highest proportion of government-owned LTCFs, accounting for 20% of the state’s LTCFs (compared with <10% in other states),^43^ and 80% of government LTCFs in this study. State legislation requires all government LTCFs to meet nurse-to-resident ratios and report quality indicators on high-risk care areas, including pressure injuries, falls and fractures, and polypharmacy (use of nine or more medicines).^44^ The matching process also reduced the sample sizes for comparison, as only residents with comparable characteristics across ownership types were retained. This limited our capacity to estimate average treatment effects and allowed us to only examine the average treatment effect on the treated. This was notable in the comparisons involving metropolitan government LTCFs where the smaller matched sample may not reflect the broader population. Our findings should also be interpreted in the context of data from 2013 to 2018, which predate changes in LTCF case mix, and shifts in ownership structures, and the COVID-19 pandemic, which may have further influenced the relationships studied. Finally, our findings may not apply to residents of LTCFs who are DVA card holders (2.5% of individuals in LTCFs) or Aboriginal and Torres Strait Islander peoples (<1% of the individuals in LTCFs),^45^ the latter excluded due to lack of Aboriginal individuals’ leadership in this study for the inclusion of their data in these analyses.

## Conclusions and implications

Using longitudinal, person-level data, this nationwide study identified that for-profit and not-for-profit LTCF ownership compared with government ownership was associated with higher risks of hospitalisation. Higher risk of mortality in for-profit and government LTCFs compared with not-for-profits was also observed in certain geographical regions. Most significant outcome differences were observed in inner regional LTCFs but were generally consistent within other areas examined. While we did not examine factors such as staff, care quality and organisational culture, our detailed analyses considered personal, socioeconomic and health factors that affected the studied outcomes. With the Australian long-term care sector undergoing privatisation with a significant proportion of for-profit providers, our results highlight the importance of ongoing monitoring of ownership trends and their association with care quality and resident outcomes.

## Supplementary material

10.1136/bmjopen-2026-117917 online supplemental file 1

## Data Availability

Data may be obtained from a third party and are not publicly available.
