# Supplementary material for: Association of long-term care facility ownership and location on residents’ mortality and hospitalisations: A population-based retrospective cohort study in Australia
Source: BMJ Open. 2026 Sep 20;16(9):e117917. doi: 10.1136/bmjopen-2026-117917 (PMC13599915; doi:10.1136/bmjopen-2026-117917)
Supplement: Supplementary file 1 [file bmjopen-2026-117917.reviewer_comments.pdf]

PEER REVIEW HISTORY

BMJ Open publishes all reviews undertaken for accepted manuscripts. Reviewers are asked to complete a checklist review form and are provided with free text boxes to elaborate on their assessment. These free text comments are reproduced below.

ARTICLE DETAILS

Title (Provisional)

The association of long-term care facility ownership and location on residents' mortality and hospitalisations: A population-based retrospective cohort study in Australia

Authors

Rahja, Miia; Davies, Ling W; Jorissen, Robert N.; Crotty, Maria; Whitehead, Craig; Evans, Keith; Corlis, Megan; Meyer, Carly; Caughey, Gillian Elizabeth; Inacio, Maria C

VERSION 1 - REVIEW

**Reviewer**	**1**
**Name**	**Quail, Patrick**
**Affiliation**	**University of Calgary Cumming School of Medicine, Family Medicine**
**Date**	**27-Feb-2026**
**COI**	**None**

Thank you for the opportunity to review your manuscript. I found it intriguing and well written. My primary concern and it may also be a concern for the Editors is the absence of any attempt to match sites based on assessed medical, nursing and personal care needs in addition to the omission of any reference to the assignment process from the community/hospital based on these assessments. It may well be that LTCFs had poorer outcomes based on these unmeasured needs.So while propensity score matching does a decent job at mimicking a prospective study it is only as good as the measured co-founders.The absence or presence of dementia as a diagnosis is not a sufficient measure of need or individual risk to make any conclusions regarding ownership type. While mortality is an interesting outcome it is not nearly as useful as avoidable hospitalizations , avoidable days in hospital, deaths in hospital etc. It may be sufficient to add this as a limitation but I think it would strengthen your paper to produce some evidence on the comparability of homes that would more comprehensively control for ownership.

**Reviewer**	**2**
**Name**	**Wagg, Adrian**
**Affiliation**	**Univ Alberta, medicine**
**Date**	**29-May-2026**
**COI**	**None**

eviewer's report: The impact of long-term care facility ownership and location on residents' mortality and hospitalisations

The aim of this retrospective, propensity score-matched, cohort study was to assess whether LTCF ownership was associated with risks of mortality, emergency department (ED) presentations, unplanned hospitalisations, and hospital days during the first 12-months after LTCF entry, and whether these associations varied by geographical region in Australia.

Abstract: The abstract is a concise accurate report of the study and the conclusions justified based upon that presented results. I wonder if, in method the authors might note whether their hazard ratios are adjusted?

Introduction

A well written, well supported succinct paragraph which outlines the problem and defines the research question well stop I have no suggestions for change

Methods.

If anything, the data are now old and, as the authors note the case mix in nursing homes has drastically changed due to the influence of aging in place policies. Additionally, these data predate the ownership alterations noted in the introduction and the impact of the COVID-19 pandemic. If not already acknowledged, these are significant limitations. Hopefully, having established the data linkages more timely updates will be possible.

Please could the authors explain why indigenous peoples were excluded?

I could not see the data which noted the number of excluded individuals following propensity matching.

When calculating days spent in hospital, is there a data flag which indicates the awaiting alternative levels of care or awaiting transfer?

If analyzed by operator status this might reveal interesting differences which help illustrate the efficiencies of discharge process and observed length of stay may not all be due to acute illness.

Was there a variable for functional ability /dependency or mobility used in the covariate list? I could not see one

the statistical analysis plan is well reported and suitable given the nature of the data

Results: Well, presented with a wealth of data. The combination of text and tables is excellent

Discussion: well written and argued with appropriate attempts at explanatory statements. strengths and limitations suitably addressed

---

VERSION 1 - AUTHOR RESPONSE

Reviewer 1 comments to author:

**Comment**	**Response to comment**	**Location on manuscript**
Thank you for the opportunity to review your manuscript. I found it intriguing and well written.	Thank you.	n/a
My primary concern and it may also be a concern for the Editors is the absence of any attempt to match sites based on assessed medical, nursing and personal care needs in addition to the omission of any reference to the assignment process from the community/hospital based on these assessments. It may well be that LTCFs had poorer outcomes based on these unmeasured needs. So while propensity score matching does a decent job at mimicking a prospective study it is only as good as the measured co-founders.	Thank you for this important suggestion. While we have attempted to capture individuals' care needs through the inclusion of individual characteristics including their detailed history of service hospitalisation prior to entering care, we agree that consideration of residents' functional care needs is relevant when comparing LTCFs and we considered this during the development of our analysis.<br>However, there are important limitations with the available national data sources that captured resident's care level needs in Australia during this study period. Specifically, during our study period, in Australia, care needs in LTCFs were assessed using the Aged Care Funding Instrument (ACFI). Government-commissioned evaluations, including the Alternative Aged Care Assessment, Classification System and Funding Models Final Report (Australian Health Services Research Institute, 2017), identified limitations of the ACFI approach and the need for substantial redesign of assessment and classification methods for funding and care need measurement. Prior work has also highlighted variability in how ACFI assessments are applied across providers- particularly related to ownership type- raising concerns about systematic differences in reporting by facility characteristics (Loggie et al., 2021). Given these constraints, we were concerned that the inclusion of these measures may introduce bias rather than improve comparability between LTCFs. For these reasons, and in the absence of an unbiased, nationally consistent measure of	Strengths and limitations

	residents' care needs suitable for this purpose, we did not include these variables in the primary analyses.<br>We agree this is an important limitation and have now expanded the discussion of this issue in the manuscript limitations section.	
The absence or presence of dementia as a diagnosis is not a sufficient measure of need or individual risk to make any conclusions regarding ownership type.	Noted, thank you. We agree that the absence or presence of a dementia diagnosis alone is not a sufficient proxy for care need or individual risk. However, the study does not rely on dementia status as a sole measure of need or as a basis for conclusions regarding ownership type, and also accounts for other health conditions, including prior health service use (e.g., primary care utilisation).	n/a
While mortality is an interesting outcome it is not nearly as useful as avoidable hospitalizations, avoidable days in hospital, deaths in hospital etc. It may be sufficient to add this as a limitation but I think it would strengthen your paper to produce some evidence on the comparability of homes that would more comprehensively control for ownership.	Thank you for this helpful point. We agree that mortality alone may be less sensitive than other indicators, such as potentially avoidable hospitalisations, for capturing differences. However, in addition to mortality, we did examine ED presentations, unplanned hospitalisations, and days spent in hospital, which provide a broader assessment of health service use and outcomes. We have clarified this in the manuscript and acknowledge in the limitations that not all outcomes (e.g., avoidable hospitalisations or place of death) were examined as follows: *While we included multiple outcomes (mortality, ED presentations, unplanned hospitalisations and hospital length of stay), these measures may not fully capture differences in care quality across LTCFs. More specific indicators such as potentially avoidable hospitalisations or place of death were not examined.*	Strengths and limitations

Reviewer 2 comments to author:

The aim of this retrospective, propensity score-matched, cohort study was to assess whether LTCF ownership was associated with risks of mortality, emergency department (ED) presentations, unplanned hospitalisations, and hospital days during the first 12-months after LTCF entry, and whether these associations varied by geographical region in Australia.

**Comment**	**Response to comment**	**Location on manuscript**
**Abstract**:<br>The abstract is a concise accurate report of the study and the conclusions justified based upon that presented results. I wonder	Thank you for picking this up. We have added that we report 'adjusted' hazard ratios in the methods section of the abstract.	Abstract

if, in method the authors might note whether their hazard ratios are adjusted?		
**Introduction**<br>A well written, well supported succinct paragraph which outlines the problem and defines the research question well stop I have no suggestions for change	Thank you.	n/a
**Methods**<br>If anything, the data are now old and, as the authors note the case mix in nursing homes has drastically changed due to the influence of aging in place policies. Additionally, these data predate the ownership alterations noted in the introduction and the impact of the COVID-19 pandemic. If not already acknowledged, these are significant limitations. Hopefully, having established the data linkages more timely updates will be possible.	These are some great points. We have added an acknowledgement of these limitations also in our discussion as follows: *Our findings should also be interpreted in the context of data from 2013–2018, which predate some of earlier-mentioned changes in LTCF case mix associated with ageing-in-place policies, as well as shifts in ownership structures. The data also precede the COVID-19 pandemic, which may have further influenced service delivery and resident outcomes, potentially limiting applicability to current practice.*	Strengths and limitations
Please could the authors explain why indigenous peoples were excluded?	These peoples were excluded from our study as our team abides by the South Australian Aboriginal Health Research Accord and any study or analysis that includes their data requires leadership and governance by indigenous individuals, which were not included in this study. We added a clarification regarding this in the strengths and limitations section of our discussion as follows; *…or Aboriginal and Torres Strait Islander peoples (<1% of the individuals in LTCFs) (43) as these groups were excluded due to lack of Aboriginal individuals' leadership in this study for the inclusion of their data in these analyses.*	Strengths and limitations
I could not see the data which noted the number of excluded individuals following propensity matching.	The data is not as obviously presented, but visible from the comparison of before and after matching data presented in the Supplementary Tables S2-S10.	Supplementary file
When calculating days spent in hospital, is there a data flag which indicates the awaiting alternative levels of care or awaiting transfer? If analyzed by operator status this might reveal interesting differences which	No, we do not have this data.<br>We do agree, however, that this would be interesting.	n/a

help illustrate the efficiencies of discharge process and observed length of stay may not all be due to acute illness.		
Was there a variable for functional ability /dependency or mobility used in the covariate list? I could not see one<br>the statistical analysis plan is well reported and suitable given the nature of the data	This appears to be in relation to the comment also provided by the other reviewer. Consideration of residents' functional ability/mobility / care needs would be highly relevant when comparing LTCFs and we considered this during the development of our analysis. As we also responded to the other reviewer, while we have attempted to capture individuals' care needs through the inclusion of individual characteristics including their detailed history of service hospitalisation prior to entering care, we agree that consideration of residents' functional care needs is relevant when comparing LTCFs and we considered this during the development of our analysis.<br>However, there are important limitations with the available national data sources that captured resident's care level needs in Australia during this study period. Specifically, during our study period, in Australia, care needs in LTCFs were assessed using the Aged Care Funding Instrument (ACFI). Government-commissioned evaluations, including the Alternative Aged Care Assessment, Classification System and Funding Models Final Report (Australian Health Services Research Institute, 2017), identified limitations of the ACFI approach and the need for substantial redesign of assessment and classification methods for funding and care need measurement. Prior work has also highlighted variability in how ACFI assessments are applied across providers- particularly related to ownership type- raising concerns about systematic differences in reporting by facility characteristics (Loggie et al., 2021). Given these constraints, we were concerned that the inclusion of these measures may introduce bias rather than improve comparability between LTCFs. For these reasons, and in the absence of an unbiased, nationally consistent measure of residents' care needs suitable for this purpose, we did	n/a

	not include these variables in the primary analyses.<br>We agree this is an important limitation, and have now expanded the discussion of this issue in the manuscript limitations section.	
**Results**<br>Well, presented with a wealth of data. The combination of text and tables is excellent	Thank you.	n/a
**Discussion**<br>Well written and argued with appropriate attempts at explanatory statements. strengths and limitations suitably addressed	Thank you.	n/a

VERSION 2 - REVIEW

**Reviewer**	**2**
**Name**	**Wagg, Adrian**
**Affiliation**	**Univ Alberta, medicine**
**Date**	**15-Aug-2026**
**COI**	

the authors have made thoughtful revisions which have strengthened their paper. I have no further comments
