# Supplementary material for: Association of long-term care facility ownership and location on residents’ mortality and hospitalisations: A population-based retrospective cohort study in Australia
Source: BMJ Open. 2026 Sep 20;16(9):e117917. doi: 10.1136/bmjopen-2026-117917 (PMC13599915; doi:10.1136/bmjopen-2026-117917)
Supplement: Supplementary file 2 [file bmjopen-16-9-s001.docx]

# Table S1. Medicare Benefits Schedule subsidised health care services included in analysis

| Health Care Services Exposures | | | |
| --- | --- | --- | --- |
| MBS Ascertained | **MBS Groups** | **MBS Group Description** | **MBS items** |
| General Attendances | | | |
| GP/Medical practitioner attendances | A01  A02  A35 | GP attendances  Non-referred attendance to medical practitioner  Medical services at residential aged care facilities | 3, 4, 23, 24, 36, 37, 44, 47  52, 53, 54 ,57, 58, 59, 60, 65  20, 35, 43, 51, 92, 93, 95, 96, 183, 188, 202, 212, 90020, 90035, 90043, 90051, 90092, 90093, 90095, 90096,  90183, 90188, 90202, 90212 |
| After-hours attendances | A22  A23 | GP after-hours attendance  Non-referred after-hours attendance with medical practitioners | 5000, 5003, 5010, 5020, 5023, 5028, 5040, 5043, 5049, 5060, 5063, 5067  5200, 5203, 5207, 5208, 5220, 5223, 5227, 5228, 5260, 5263, 5265, 5267 |
| Urgent attendances after-hours | A11 | Urgent GP/Medical practitioner attendance after-hours | 585, 588, 591, 594, 597, 598, 599, 600 |
| Health Assessments / Management Plans | | | |
| Health assessments | A14 | GP/Medical practitioner health assessments | 224, 225, 226, 227, 701, 703, 705, 707 |
| Management plans | A15 | GP/Medical practitioner management plan attendances/team care arrangements and multidisciplinary care plans | 229, 230, 231, 232, 233, 235, 236, 237, 238, 239, 240, 243, 244, 721, 723, 729, 731, 732, 735, 739, 743, 747, 750, 758, 871, 872 |
| Selected Specialist Attendances | | | |
| Pain medicine | A24 | Pain medicine attendances | 2799, 2801, 2806, 2814, 2820, 2824, 2832, 2840, 2946, 2949, 2954, 2958, 2972, 2974, 2978, 2984, 2988, 2996, 3000 |
| Geriatric medicine | A28 | Geriatric medicine attendances | 141, 143, 145, 147, 149 |
| Abbreviations: MBS, Medicare benefits schedule; GP, general practitioner | | | |

## **Table S2.** **Long-term care facility residents’ characteristics by ownership model and by geographical remoteness, 2013-2018**

| **Geographical remoteness** | **Metropolitan** | | | **Inner regional** | | | **Outer regional, remote and very remote** | | |
| --- | --- | --- | --- | --- | --- | --- | --- | --- | --- |
|  | **(N=141509, 69.0%)** | | | **(N=49402, 24.1%)** | | | **(N=14168, 6.9%)** | | |
| **Ownership model** | **Government** | **Not-for-profit** | **For-profit** | **Government** | **Not-for-profit** | **For-profit** | **Government** | **Not-for-profit** | **For-profit** |
| N (%) | 1575 (1.11) | 65334 (46.2) | 74600 (52.7) | 5294 (10.7) | 30882 (62.5) | 13226 (26.8) | 2092 (14.8) | 8928 (63.0) | 3148 (22.2) |
| **Sex (N, %)** | | | | | | | | | |
| Female | 767 (48.7) | 40600 (62.4) | 45119 (60.5) | 3057 (57.7) | 18811 (60.9) | 7634 (57.7) | 1225 (58.6) | 5304 (59.4) | 1782 (56.6) |
| Male | 808 (51.3) | 24734 (37.9) | 29481 (39.5) | 2237 (42.3) | 12071 (39.1) | 5592 (42.3) | 867 (41.4) | 3624 (40.6) | 1366 (43.4) |
| **Age, median (IQR)** | 83 (76-88) | 85 (80-90) | 85 (80-89) | 84 (79-89) | 85 (80-89) | 85 (79-89) | 85 (80-89) | 85 (79-89) | 84 (79-89) |
| **Partner status; Yes, partner (N, %)** | 675 (42.9) | 23185 (35.5) | 28230 (37.8) | 2086 (39.4) | 11546 (37.4) | 5366 (40.6) | 784 (37.5) | 3091 (34.6) | 1257 (39.9) |
| **Number of comorbidities (N, %)** | | | | | | | | | |
| 0-1 | 163 (10.3) | 5622 (8.60) | 5893 (7.90) | 444 (8.40) | 2319 (7.50) | 1035 (7.800) | 158 (7.60) | 793 (8.90) | 270 (8.60) |
| 2 to 3 | 298 (18.9) | 11922 (18.2) | 13278 (17.8) | 952 (18.0) | 5421 (17.6) | 2448 (18.5) | 353 (16.9) | 1715 (19.2) | 567 (18.0) |
| 4 to 5 | 398 (25.3) | 18162 (27.8) | 20166 (27.0) | 1430 (27.0) | 8792 (28.5) | 3590 (27.1) | 589 (28.2) | 2533 (28.4) | 868 (27.6) |
| 6 to 7 | 393 (25.0) | 16253 (24.9) | 19041 (25.5) | 1328 (25.1) | 7723 (25.0) | 3377 (25.5) | 521 (24.9) | 2190 (24.5) | 808 (25.7) |
| 8+ | 323 (20.5) | 13375 (20.5) | 16222 (21.7) | 1140 (21.5) | 6627 (21.5) | 2776 (21.0) | 471 (22.5) | 1697 (19.0) | 635 (20.2) |
| **Health conditions (N, %)** | | | | | | | | | |
| Dementia | 840 (53.3) | 34411 (52.7) | 39798 (53.3) | 2618 (49.5) | 14402 (46.6) | 6634 (50.2) | 973 (46.5) | 4223 (47.3) | 1542 (49.0) |
| History of delirium | 144 (9.10) | 4441 (6.80) | 5819 (7.80) | 245 (4.60) | 1438 (4.70) | 778 (5.90) | 80 (3.80) | 340 (3. 80) | 165 (5.20) |
| Malnutrition | 125 (7.90) | 4264 (6.50) | 5105 (6.80) | 365 (6.90) | 1617 (5.20) | 784 (5.90) | 121 (5.80) | 460 (5.20) | 203 (6.40) |
| Incontinence | 414 (26.3) | 21010 (32.2) | 25612 (34.3) | 1475 (27.9) | 9034 (29.3) | 4435 (33.5) | 533 (25.5) | 2460 (27.6) | 1130 (35.9) |
| Pressure injuries | 56 (3.60) | 1812 (2.80) | 1959 (2.60) | 135 (2.60) | 765 (2.50) | 395 (3.00) | 66 (3.20) | 192 (2.20) | 80 (2.50) |
| Depression | 324 (20.6) | 14168 (21.7) | 15855 (21.3) | 1066 (20.1) | 6436 (20.8) | 2774 (21.0) | 441 (21.1) | 1989 (22.3) | 724 (23.0) |
| **SEIFA RSD Quintile (N, %)** | | | | | | | | | |
| 1 (Most disadvantaged) | 232 (14.7) | 7273 (11.1) | 9511 (12.7) | 1389 (26.2) | 9158 (29.7) | 3361 (25.4) | 611 (29.2) | 3126 (35.0) | 788 (25.0) |
| 2 | 244 (15.5) | 8628 (13.2) | 9560 (12.8) | 1760 (33.2) | 9093 (29.4) | 3245 (24.5) | 862 (41.2) | 3374 (37.8) | 1590 (50.5) |
| 3 | 247 (15.7) | 13082 (20.0) | 12677 (17.0) | 1142 (21.6) | 8168 (26.4) | 3243 (24.5) | 357 (17.1) | 1780 (19.9) | 426 (13.5) |
| 4 | 363 (23.0) | 13852 (21.2) | 17677 (23.7) | 461 (8.70) | 2169 (7.00) | 1506 (11.4) | 145 (6.90) | 340 (3.80) | 182 (5.80) |
| 5 (Least disadvantaged) | 399 (25.3) | 20866 (31.9) | 23243 (31.2) | 338 (6.40) | 1839 (6.00) | 1510 (11.4) | 52 (2.50) | 194 (2.20) | 83 (2.60) |
| Missing | 90 (5.70) | 1633 (2.50) | 1932 (2.60) | 204 (3.90) | 455 (1.50) | 361 (2.70) | 65 (3.10) | 114 (1.30) | 79 (2.50) |
| **Medications (90 days)^1^** | | | | | | | | | |
| Median^2^ (IQR) | 8 (5-11) | 7 (5-11) | 8 (5-11) | 8 (5-11) | 8 (5-11) | 8 (5-11) | 8 (5-11) | 7 (4-10) | 7 (5-11) |
| High sedative load rating (>=3) (N, %) | 483 (30.7) | 16594 (25.4) | 21299 (28.5) | 1484 (28.0) | 8665 (28.1) | 3863 (29.2) | 643 (30.7) | 2376 (26.6) | 921 (29.3) |
| **Hospital encounters (365 days)^1,3^ (N, %)** | | | | | | | | | |
| Any hospitalisations |  |  |  |  |  |  |  |  |  |
| 0 | 180 (11.4) | 13171 (20.2) | 12703 (17.0) | 773 (14.6) | 6407 (20.7) | 2641 (20.0) | 318 (15.2) | 1686 (18.9) | 679 (21.6) |
| 1 | 564 (35.8) | 20805 (31.8) | 23762 (31.9) | 1763 (33.3) | 9635 (31.2) | 4182 (31.6) | 634 (30.3) | 2621 (29.4) | 965 (30.7) |
| 2 to 4 | 641 (40.7) | 24909 (38.1) | 29842 (40.0) | 2204 (41.6) | 11869 (38.4) | 5150 (38.9) | 897 (42.9) | 3680 (41.2) | 1202 (38.2) |
| 5+ | 190 (12.1) | 6449 (9.90) | 8293 (11.1) | 554 (10.5) | 2971 (9.60) | 1253 (9.50) | 243 (11.6) | 941 (10.5) | 302 (9.60) |
| **ED presentations** | | | | | | | | | |
| 0 | 293 (18.6) | 18298 (28.0) | 20005 (26.8) | 1782 (33.7) | 8031 (26.0) | 3524 (26.6) | 781 (37.3) | 2290 (25.6) | 756 (24.0) |
| 1 | 518 (32.9) | 19105 (29.2) | 21844 (29.3) | 1497 (28.3) | 8255 (26.7) | 3554 (26.9) | 499 (23.9) | 2031 (22.7) | 771 (24.5) |
| 2 to 4 | 595 (37.8) | 22658 (34.7) | 26468 (35.5) | 1578 (29.8) | 11209 (36.3) | 4762 (36.0) | 588 (28.1) | 3226 (36.1) | 1231 (39.1) |
| 5+ | 169 (10.7) | 5273 (8.10) | 6283 (8.40) | 437 (8.30) | 3387 (11.0) | 1386 (10.5) | 224 (10.7) | 1381 (15.5) | 390 (12.4) |
| **Unplanned hospitalisations** | | | | | | | | | |
| 0 | 280 (17.8) | 17161 (26.3) | 17468 (23.4) | 1203 (22.7) | 8815 (28.5) | 3614 (27.3) | 473 (22.6) | 2142 (24.0) | 835 (26.5) |
| 1 | 639 (40.6) | 23744 (36.3) | 27427 (36.8) | 2083 (39.3) | 10923 (35.4) | 4841 (36.6) | 725 (34.7) | 2963 (33.2) | 1066 (33.9) |
| 2 to 4 | 562 (35.7) | 21489 (32.9) | 25876 (34.7) | 1775 (33.5) | 9808 (31.8) | 4208 (31.8) | 762 (36.4) | 3263 (36.5) | 1083 (34.4) |
| 5+ | 94 (6.00) | 2940 (4.50) | 3829 (5.10) | 233 (4.40) | 1336 (4.30) | 563 (4.30) | 132 (6.30) | 560 (6.30) | 164 (5.20) |
| **Potentially preventable hospitalisations** | | | | | | | | | |
| 0 | 1186 (75.3) | 50173 (76.8) | 56296 (75.5) | 3931 (74.3) | 23471 (76.0) | 10099 (76.4) | 1554 (74.3) | 6570 (73.6) | 2408 (76.5) |
| 1 | 284 (18.0) | 10817 (16.6) | 12765 (17.1) | 928 (17.5) | 5089 (16.5) | 2141 (16.2) | 348 (16.6) | 1607 (18.0) | 517 (16.4) |
| 2 or more | 105 (6.70) | 4344 (6.70) | 5539 (7.40) | 435 (8.20) | 2322 (7.50) | 986 (7.50) | 190 (9.10) | 751 (8.40) | 223 (7.00) |
| **Days in hospital (if hospitalised)** | | | | | | | | | |
| 0 | 402 (25.5) | 25369 (38.8) | 26392 (35.4) | 1310 (24.7) | 13021 (42.2) | 4569 (34.6) | 748 (35.8) | 5037 (56.4) | 1619 (51.4) |
| 1 to 30 | 362 (23.0) | 17987 (27.5) | 20900 (28.0) | 1574 (29.7) | 9542 (30.9) | 4495 (34.0) | 622 (29.7) | 2251 (25.2) | 897 (28.5) |
| 31 to 90 | 513 (32.6) | 16997 (26.0) | 21169 (28.4) | 1692 (32.0) | 6552 (21.2) | 3303 (25.0) | 494 (23.6) | 1328 (14.9) | 510 (16.2) |
| 91+ | 293 (18.9) | 4981 (7.60) | 6139 (8.20) | 718 (13.6) | 1767 (5.70) | 859 (6.50) | 228 (10.9) | 312 (3.50) | 122 (3.90) |
| **Hospital encounters (30 days)^1,3^ (N, %)** | | | | | | | | | |
| Any Hospitalisations | 640 (40.6) | 22061 (33.8) | 25820 (34.6) | 2132 (40.3) | 10450 (33.8) | 4406 (33.3) | 639 (30.5) | 2912 (32.6) | 850 (27.0) |
| ED presentations | 411 (26.1) | 13513 (20.7) | 16010 (21.5) | 970 (18.3) | 7168 (23.2) | 3025 (22.9) | 301 (14.4) | 1983 (22.2) | 639 (20.3) |
| **Primary health care encounters (365 days) ^1,3^ (N, %)** | | | | | | | | | |
| General attendances^4^ |  |  |  |  |  |  |  |  |  |
| 0 | 54 (3.40) | 881 (1.40) | 1073 (1.40) | 84 (1.60) | 339 (1.10) | 143 (1.10) | 29 (1.40) | 144 (1.60) | 48 (1.50) |
| 1 to 4 | 243 (15.4) | 6709 (10.3) | 7844 (10.5) | 605 (11.4) | 3117 (10.1) | 1320 (10.0) | 186 (8.90) | 1057 (11.8) | 370 (11.8) |
| 5 to 9 | 441 (28.0) | 15964 (24.4) | 17624 (23.6) | 1286 (24.3) | 7229 (23.4) | 3151 (23.8) | 409 (19.6) | 2133 (23.9) | 1924(61.1) |
| 10+ | 837 (53.1) | 41780 (64.0) | 48059 (64.4) | 3319 (62.7) | 20197 (65.4) | 8612 (65.1) | 1468 (70.2) | 5594 (62.7) | 806 (25.6) |
| Any after-hours attendances | 570 (36.1) | 21551 (33.1) | 33526 (44.9) | 1195 (22.6) | 6160 (19.9) | 3122 (23.6) | 466 (22.3) | 1612 (18.1) | 945 (30.0) |
| Any urgent after-hour attendances | 410 (26.0) | 14900 (22.8) | 21430 (28.7) | 924 (17.4) | 2865 (9.30) | 1347 (10.2) | 319 (15.2) | 910 (10.2) | 372 (11.8) |
| Any health assessments | 437 (27.8) | 22475 (34.4) | 25816 (34.6) | 1321 (25.0) | 10092 (32.7) | 4092 (30.9) | 577 (27.6) | 2716 (30.4) | 950 (30.2) |
| Chronic disease management plans | 729 (46.3) | 35256 (54.0) | 38693 (53.2) | 2320 (43.8) | 16970 (55.0) | 6888 (52.1) | 863 (41.3) | 4287 (48.0) | 1438 (45.7) |
| Pain medicine attendances | 102 (6.50) | 7699 (11.8) | 7527 (10.1) | 285 (5.40) | 2382 (7.70) | 916 (6.90) | 65.0 (3.10) | 484 (5.40) | 182 (5.80) |
| Geriatric medicine attendances | 91 (5.80) | 7410 (11.3) | 7172 (9.60) | 262 (5.00) | 2261 (7.30) | 858 (6.50) | 56.0 (2.70) | 434 (4.90) | 163 (5.20) |
| Respite prior to LTCF entry, any (N, %) | 418 (26.5) | 31400 (48.1) | 37274 (50.0) | 2348 (44.4) | 17136 (55.5) | 7525 (56.9) | 1296 (62.0) | 5094 (57.1) | 2092 (66.5) |
| Entry immediately following respite | 235 (14.9) | 24765 (37.9) | 30845 (41.3) | 1608 (30.4) | 13362 (43.3) | 6156 (46.5) | 1044 (49.9) | 4121 (46.2) | 1795 (57.0) |
| **State of service (N, %)** | | | | | | | | | |
| New South Wales | 77 (4.90) | 33714 (51.6) | 26761 (35.9) | 525 (9.90) | 15961 (51.7) | 4976 (37.6) | 237 (11.3) | 3945 (44.2) | 744 (23.6) |
| Queensland | 234 (14.9) | 14872 (22.8) | 16465 (22.1) | 578 (10.9) | 7873 (25.5) | 2248 (17.0) | 492 (23.5) | 4057 (45.4) | 1195 (38.0) |
| Victoria | 1264 (80.3) | 16748 (25.6) | 31374 (42.1) | 4191 (79.2) | 7048 (22.8) | 6002 (45.4) | 1363 (65.2) | 926 (10.4) | 1209 (38.4) |
| **Year of care entry (N, %)** | | | | | | | | | |
| 2013 | 217 (13.8) | 10087 (15.4) | 10166 (13.6) | 789 (14.9) | 4466 (14.5) | 1812 (13.7) | 318 (15.2) | 1366 (15.3) | 429 (13.6) |
| 2014 | 219 (13.9) | 9771 (15.0) | 11816 (15.8) | 900 (17.0) | 4691 (15.2) | 1927 (14.6) | 327 (15.6) | 1341 (15.0) | 471 (15.0) |
| 2015 | 286 (18.2) | 10438 (16.0) | 12420 (16.6) | 958 (18.1) | 5094 (16.5) | 2423 (18.3) | 382 (18.3) | 1401 (15.7) | 554 (17.6) |
| 2016 | 280 (17.8) | 11305 (17.3) | 12432 (16.7) | 866 (16.4) | 5188 (16.8) | 2378 (18.0) | 320 (15.3) | 1478 (16.6) | 595 (18.9) |
| 2017 | 286 (18.2) | 11860 (18.2) | 13667 (18.3) | 893 (16.9) | 5695 (18.4) | 2273 (17.2) | 345 (16.5) | 1677 (18.8) | 548 (17.4) |
| 2018 | 287 (18.2) | 11873 (18.2) | 14099 (18.9) | 888 (16.8) | 5748 (18.6) | 2413 (18.2) | 400 (19.1) | 1665 (18.6) | 551 (17.5) |
| Notes: 1, days prior to LTCF entry; 2, number of unique medications; 3, obtained using the Medicare Benefits Schedule subsidised services in the year prior to LTCF entry (see supplementary Table S1 for detail); 4, general practitioner and medical practitioner attendances.  Abbreviations: ADL, activities of daily living; ED, emergency department; IQR, interquartile range; LTCF, long term care facility, SEIFA, Socio-Economic Indexes for Areas; RSD, relative socio-economic disadvantage index. | | | | | | | | | |

# Table S3. Long-term care facility residents’ characteristics, not-for-profit vs for-profit in metropolitan regions, before and after matching

|  | **Before matching** | | | **After matching^2^** | | |
| --- | --- | --- | --- | --- | --- | --- |
|  | **Not-for-profit**  **N=65334**  **n (% of N)** | **For-profit**  **N=74600**  **n (% of N)** | **ASMD** | **Not-for-profit**  **N=57889**  **n (% of N)** | **For-profit**  **N=57889**  **n (% of N)** | **ASMD** |
| **Male (N, %)** | 24734 (37.9) | 29481 (39.5) | 0.03 | 22559 (39.0) | 22672 (39.2) | 0.00 |
| **Age (Mean, SD)** | 84.5 (7.22) | 84.3 (7.23) | 0.04 | 84.4 (7.25) | 84.3 (7.24) | 0.01 |
| **Partner status; Yes, partner (N, %)** | 23185 (35.5) | 28230 (37.8) | 0.05 | 21262 (36.7) | 21418 (37.0) | 0.01 |
| **Health conditions (N, %)**  Dementia  History of delirium  Depression  Incontinence  Pressure injuries  Malnutrition | 34411 (52.7)  4441 (6.80)  14168 (21.7)  21010 (32.2)  1812 (2.80)  4264 (6.50) | 39798 (53.3)  5819 (7.80)  15855 (21.3)  25612 (34.3)  1959 (2.60)  5105 (6.80) | 0.01  0.04  0.01  0.05  0.01  0.01 | 30957 (53.5)  4070 (7.00)  12176 (21.0)  19165 (33.1)  1566 (2.70)  3798 (6.60) | 30921 (53.4)  4402 (7.60)  12204 (21.1)  19291 (33.3)  1580 (2.70)  3835 (6.60) | 0.00  0.02  0.00  0.01  0.00  0.00 |
| **SEIFA RSD Quintile (N, %)**  1 (Most disadvantaged)  2  3  4  5 (Least disadvantaged)  Missing | 7273 (11.1)  8628 (13.2)  13082 (20.0)  13852 (21.2)  20866 (31.9)  1633 (2.50) | 9511 (12.7)  9560 (12.8)  12677 (17.0)  17677 (23.7)  23243 (31.2)  1932 (2.60) | 0.10 | 7082 (12.2)  7410 (12.8)  10146 (17.5)  12949 (22.4)  18731 (32.4)  1571 (2.70) | 7227 (12.5)  7601 (13.1)  10316 (17.8)  12853 (22.2)  18329 (31.7)  1563 (2.70) | 0.02 |
| **Medications (90 days)^1^ (Mean, SD)** | 7.98 (4.71) | 8.18 (4.80) | 0.04 | 7.97 (4.72) | 8.03 (4.79) | 0.01 |
| **Sedative load (90 days)^1^ (Mean, SD)** | 1.64 (1.86) | 1.78 (1.95) | 0.08 | 1.68 (1.89) | 1.70 (1.90) | 0.01 |
| **Number of comorbidities (Mean, SD)** | 5.25 (2.72) | 5.37 (2.73) | 0.04 | 5.27 (2.73) | 5.29 (2.73) | 0.01 |
| **Hospital encounters (365 days^1^; Mean, SD)**  ED Presentations  Hospitalisations  Days in hospital | 1.78 (2.23)  2.43 (7.19)  29.0 (71.4) | 1.81 (2.25)  2.66 (7.80)  30.8 (57.7) | 0.02  0.03  0.03 | 1.82 (2.28)  2.56 (7.59)  29.7 (74.9) | 1.81 (2.18)  2.51 (6.94)  29.0 (49.5) | 0.01  0.01  0.01 |
| **Primary health care (365 days^1^; Mean, SD)**  General attendances  Urgent after hour attendances  Health assessments  Chronic disease management plans  Pain medicine attendances  Geriatric medicine attendances | 14.7 (11.2)  0.40 (1.24)  0.37 (0.55)  1.36 (1.64)  0.17 (0.58)  0.16 (0.50) | 14.7 (10.9)  0.52 (1.46)  0.38 (0.56)  1.34 (1.65)  0.15 (0.53)  0.14 (0.47) | <0.001  0.09  0.01  0.01  0.04  0.05 | 14.7 (11.2)  0.43 (1.32)  0.38 (0.55)  1.34 (1.63)  0.17 (0.58)  0.15 (0.48) | 14.7 (11.0)  0.44 (1.23)  0.38 (0.56)  1.34 (1.65)  0.16 (0.55)  0.15 (0.49) | 0.00  0.01  0.01  0.00  0.01  0.00 |
| **Respite prior to LTCF entry, any (N, %)** | 31400 (48.1) | 37274 (50.0) | 0.04 | 28783 (49.7) | 28802 (49.8) | 0.00 |
| **State of service (N, %)**  New South Wales  Queensland  Victoria | 33714 (51.6)  14872 (22.8)  16748 (25.6) | 26761 (35.9)  16465 (22.1)  31374 (42.1) | 0.38 | 22670 (46.1)  14471 (25.0)  16748 (28.9) | 26166 (45.2)  14025 (24.2)  17698 (30.6) | 0.04 |
| **Year of care entry (N, %)**  2013  2014  2015  2016  2017  2018 | 10087 (15.4)  9771 (15.0)  10438 (16.0)  11305 (17.3)  11860 (18.2)  11873 (18.2) | 10166 (13.6)  11816 (15.8)  12420 (16.6)  12432 (16.7)  13667 (18.3)  14099 (18.9) | 0.06 | 8246 (14.2)  8913 (15.4)  9473 (16.4)  9826 (16.7)  10597 (18.3)  10834 (18.7) | 8319 (14.4)  8940 (15.4)  9418 (16.3)  9826 (16.6)  10557 (18.2)  10829 (18.7) | 0.01 |
| Notes: 1, days prior to LTCF entry. 2, inverse probability of treatment weighting was applied, with weights truncated at values below the 1st percentile or above the 99th percentile.  Abbreviations: ASMD; average standardised mean difference; SD, standard deviation; SEIFA, Socio-Economic Indexes For Areas; RSD, relative socio-economic disadvantage; LTCF, long-term care facility; ED, emergency department; GP, general practitioner; NSW, New South Wales; QLD, Queensland; VIC, Victoria | | | | | | |

# Table S4. Long-term care facility residents’ characteristics, not-for-profit vs for-profit in inner regional areas, before and after matching

|  | **Before matching** | | | **After matching^2^** | | |
| --- | --- | --- | --- | --- | --- | --- |
|  | **Not-for-profit**  **N=30882**  **n (% of N)** | **For-profit**  **N=13226**  **n (% of N)** | **ASMD** | **Not-for-profit**  **N=12306**  **n (% of N)** | **For-profit**  **N=12306**  **n (% of N)** | **ASMD** |
| **Male (N, %)** | 12071 (39.1) | 5592 (42.3) | 0.07 | 5106 (41.5) | 5168 (42.0) | 0.01 |
| **Age (Mean, SD)** | 84.3 (7.07) | 83.7 (7.25) | 0.08 | 83.7 (7.13) | 83.8 (7.24) | 0.01 |
| **Partner status; Yes, partner (N, %)** | 11546 (37.4) | 5366 (40.6) | 0.07 | 4700 (38.2) | 5003 (40.7) | 0.05 |
| **Health conditions**  Dementia  History of delirium  Depression  Incontinence  Pressure injuries  Malnutrition | 14402 (46.6)  1438 (4.70)  6436 (20.8)  9034 (29.3)  765 (2.50)  1617 (5.20) | 6634 (50.2)  778 (5.90)  2774 (21.0)  4435 (33.5)  395 (3.00)  784 (5.90) | 0.07  0.06  0.00  0.09  0.03  0.03 | 6063 (49.3)  670 (5.40)  2574 (20.9)  4092 (33.3)  344 (2.80)  702 (5.70) | 6128 (49.8)  687 (5.60)  2534 (20.6)  4066 (33.0)  360 (2.90)  709 (5.80) | 0.01  0.01  0.01  0.00  0.01  0.00 |
| **SEIFA RSD quintile**  1 (Most disadvantaged)  2  3  4  5 (Least disadvantaged)  Missing | 9158 (29.7)  9093 (29.4)  8168 (26.4)  2169 (7.00)  1839 (6.00)  455 (1.50) | 3361 (25.4)  3245 (24.5)  3243 (24.5)  1506 (11.4)  1510 (11.4)  361 (2.80) | 0.29 | 3336 (27.1)  3235 (26.3)  3204 (26.0)  1133 (9.20)  1095 (8.90)  303 (2.50) | 3354 (27.3)  3223 (26.2)  3169 (25.8)  1140 (9.30)  1115 (9.10)  305 (2.30) | 0.01 |
| **Medications (90 days)^1^ (Mean, SD)** | 8.11 (4.57) | 8.09 (4.62) | 0.00 | 8.05 (4.59) | 8.07 (4.59) | 0.01 |
| **Sedative load (90 days)^1^ (Mean, SD)** | 1.77 (1.92) | 1.83 (1.94) | 0.03 | 1.81 (1.95) | 1.82 (1.93) | 0.01 |
| **Comorbidities (Mean, SD)** | 5.36 (2.69) | 5.32 (2.70) | 0.02 | 5.33 (2.71) | 5.33 (2.69) | 0.00 |
| **Health service use (365 days^1^; Mean, SD)**  Hospitalisations  Length of Stay  ED Presentations | 2.30 (6.29)  24.1 (63.3)  2.04 (2.79) | 2.31 (6.09)  27.2 (54.5)  1.97 (2.35) | 0.00  0.05  0.03 | 2.37 (6.78)  26.7 (62.7)  2.03 (3.29) | 2.33 (6.28)  26.5 (55.3)  1.98 (2.36) | 0.01  0.00  0.02 |
| **Primary health care (365 days^1^; (N, %)**  General attendances  Urgent after hour attendances  Health assessments  Chronic disease management plans  Pain medicine attendances  Geriatric medicine attendances | 15.9 (12.8)  0.14 (0.65)  0.35 (0.51)  1.49 (1.77)  0.11 (0.51)  0.10 (0.37) | 15.5 (12.2)  0.15 (0.61)  0.33 (0.51)  1.36 (1.70)  0.10 (0.45)  0.09 (0.35) | 0.03  0.01  0.03  0.07  0.02  0.03 | 15.8 (12.9)  0.15 (0.64)  0.34 (0.51)  1.41 (1.74)  0.10 (0.42)  0.08 (0.35) | 15.6 (12.2)  0.15 (0.61)  0.33 (0.51)  1.37 (1.71)  0.10 (0.45)  0.09 (0.35) | 0.01  0.00  0.01  0.02  0.01  0.01 |
| **Respite prior to LTCF entry, any (N, %)** | 17136 (55.5) | 7525 (56.9) | 0.03 | 7126 (57.9) | 7201 (58.5) | 0.01 |
| **State of service (N, %)**  New South Wales  Queensland  Victoria | 15961 (51.7)  7873 (25.5)  7048 (22.8) | 4976 (37.6)  2248 (17.0)  6002 (45.4) | 0.49 | 4939 (40.1)  2280 (18.5)  5087 (41.3) | 4976 (40.4)  2248 (18.3)  5082 (41.3) | 0.01 |
| **Year of care entry (N, %)**  2013  2014  2015  2016  2017  2018 | 4466 (14.5)  4691 (15.2)  5094 (16.5)  5188 (16.8)  5695 (18.4)  5748 (18.6) | 1812 (13.7)  1927 (14.6)  2423 (18.3)  2378 (18.0)  2273 (17.2)  2413 (18.2) | 0.07 | 1727 (14.0)  1822 (14.8)  2191 (17.8)  2135 (17.3)  2127 (17.3)  2304 (18.7) | 1696 (13.8)  1782 (14.5)  2226 (18.1)  2184 (17.7)  2139 (17.4)  2279 (18.5) | 0.02 |
| Notes: 1, refers to days prior to LTCF entry; 2, inverse probability of treatment weighting was applied, with weights truncated at values below the 1st percentile or above the 99th percentile.  Abbreviations: ASMD; average standardised mean difference; SD, standard deviation; SEIFA, socio-economic indexes for areas; RSD, relative socio-economic disadvantage; LTCF, long-term care facility; ED, emergency department; GP, general practitioner; NSW, New South Wales; QLD, Queensland; VIC, Victoria | | | | | | |

# Table S5. Long-term care facility residents’ characteristics, not-for-profit vs for-profit in outer regional, remote and very remote areas, before and after matching

|  | **Before matching** | | | **After matching^2^** | | |
| --- | --- | --- | --- | --- | --- | --- |
|  | **Not-for-profit**  **N=8928**  **n (% of N)** | **For-profit**  **N=3148**  **n (% of N)** | **ASMD** | **Not-for-profit**  **N=2752**  **n (% of N)** | **For-profit**  **N=2752**  **n (% of N)** | **ASMD** |
| **Male (N, %)** | 3624 (40.6) | 1366 (43.4) | 0.06 | 1178 (42.8) | 1185 (43.1) | 0.01 |
| **Age (Mean, SD)** | 83.8 (7.10) | 83.6 (7.35) | 0.02 | 83.5 (7.27) | 83.6 (7.34) | 0.01 |
| **Partner status; Yes, partner (N, %)** | 3091 (34.6) | 1257 (39.9) | 0.11 | 1068 (38.8) | 1063 (38.6) | 0.00 |
| **Health conditions**  Dementia  History of delirium  Depression  Incontinence  Pressure injuries  Malnutrition | 4223 (47.3)  340 (3.80)  1989 (22.3)  2460 (27.6)  192 (2.20)  460 (5.20) | 1542 (49.0)  165 (5.20)  724 (23.0)  1130 (35.9)  80 (2.50)  203 (6.40) | 0.03  0.07  0.02  0.18  0.03  0.06 | 1388 (50.4)  142 (5.2)  601 (21.8)  997 (36.2)  76.0 (2.80)  154 (5.60) | 1363 (49.5)  140 (5.10)  633 (23.0)  969 (35.2)  71.0 (2.60)  167 (6.10) | 0.02  0.00  0.03  0.02  0.01  0.02 |
| **SEIFA RSD quintile**  1 (Most disadvantaged)  2  3  4  5 (Least disadvantaged)  Missing | 3126 (35.0)  3374 (37.8)  1780 (19.9)  340 (3.80)  194 (2.20)  114 (1.30) | 788 (25.0)  1590 (50.5)  426 (13.5)  182 (5.80)  83 (2.60)  79 (2.50) | 0.34 | 642 (23.3)  1456 (52.9)  388 (14.1)  141 (5.10)  67 (2.40)  58 (2.10) | 687 (25.0)  1395 (50.7)  405 (14.7)  143 (5.20)  69 (2.50)  53 (1.90) | 0.05 |
| **Medications (90 days)^1^ (Mean, SD)** | 7.66 (4.44) | 7.77 (4.45) | 0.02 | 7.7 (4.35) | 7.68 (4.4) | 0.00 |
| **Sedative load (90 days)^1^ (Mean, SD)** | 1.67 (1.84) | 1.80 (1.90) | 0.07 | 1.76 (1.87) | 1.78 (1.9) | 0.01 |
| **Comorbidities (Mean, SD)** | 5.14 (2.70) | 5.23 (2.70) | 0.03 | 5.19 (2.67) | 5.19 (2.7) | 0.00 |
| **Health service use (365 days^1^; Mean, SD)**  Hospitalisations  Length of Stay  ED Presentations | 2.30 (5.02)  16.5 (40.2)  2.45 (3.82) | 2.12 (4.58)  18.6 (49.3)  2.24. (2.72) | 0.04  0.05  0.07 | 2.00 (2.33)  15.9 (37.5)  2.28 (3.81) | 2.12 (4.77)  17.0 (50.3)  2.22 (2.67) | 0.03  0.02  0.02 |
| **Health service use (365 days^1^; Mean, SD)**  General attendances  Urgent after hour attendances  Health assessments  Chronic disease management plans  Pain medicine attendance  Geriatric medicine attendances | 15.7 (13.2)  0.17 (0.71)  0.32 (0.50)  1.28 (1.76)  0.08 (0.38)  0.06 (0.29) | 14.5 (11.7)  0.19 (0.69)  0.33 (0.52)  1.15 (1.66)  0.08 (0.40)  0.07 (0.31) | 0.10  0.03  0.01  0.07  0.01  0.02 | 15.1 (12.3)  0.20 (0.63)  0.34 (0.51)  1.25 (1.81)  0.08 (0.45)  0.06 (0.30) | 14.91(12.1)  0.19 (0.70)  0.34 (0.53)  1.21 (1.70)  0.08 (0.34)  0.07 (0.32) | 0.01  0.02  <0.001  0.02  0.01  0.02 |
| **Respite prior to LTCF entry, any (N, %)** | 5094 (57.1) | 2092 (66.5) | 0.19 | 1795 (65.2) | 1774 (64.5) | 0.02 |
| **State of service (N, %)**  New South Wales  Queensland  Victoria | 3945 (44.2)  4057 (45.4)  926 (10.4) | 744 (23.6)  1195 (38.0)  1209 (38.4) | 0.72 | 718 (26.1)  1221 (44.4)  813 (29.5) | 744 (27.0)  1169 (42.5)  839 (30.5) | 0.04 |
| **Year of care entry (N, %)**  2013  2014  2015  2016  2017  2018 | 1366 (15.3)  1341 (15.0)  1401 (15.7)  1478 (16.6)  1677 (18.8)  1665 (18.6) | 429 (13.6)  471 (15.0)  554 (17.6)  595 (18.9)  548 (17.4)  551 (17.5) | 0.10 | 357 (13.0)  409 (14.9)  475 (17.3)  518 (18.8)  480 (17.4)  513 (18.6) | 366 (13.3)  421 (15.3)  478 (17.4)  519 (18.9)  478 (17.4)  490 (17.8) | 0.03 |
| Notes: 1, refers to days prior to LTCF entry; 2, inverse probability of treatment weighting was applied, with weights truncated at values below the 1st percentile or above the 99th percentile.  Abbreviations: ASMD; average standardised mean difference; SD, standard deviation; SEIFA, socio-economic indexes for areas; RSD, relative socio-economic disadvantage; LTCF, long-term care facility; ED, emergency department; GP, general practitioner; NSW, New South Wales; QLD, Queensland; VIC, Victoria | | | | | | |

# Table S6. Long-term care facility residents’ characteristics, not-for-profit vs government in metropolitan areas, before and after matching

|  | **Before matching** | | | **After matching^2^** | | |
| --- | --- | --- | --- | --- | --- | --- |
|  | **Not-for-profit**  **N=65334**  **n (% of N)** | **Govt**  **N=1575**  **n (% of N)** | **ASMD** | **Not-for-profit**  **N=1545**  **n (% of N)** | **Govt**  **N=1545**  **n (% of N)** | **ASMD** |
| **Male (N, %)** | 24734 (37.9) | 808 (51.3) | 0.27 | 773 (50.0) | 787 (50.9) | 0.02 |
| **Age (Mean, SD)** | 84.5 (7.22) | 81.6 (8.08) | 0.38 | 81.7 (7.68) | 81.8 (8.03) | 0.01 |
| **Partner status; Yes, partner (N, %)** | 23185 (35.5) | 675 (42.9) | 0.15 | 660 (42.7) | 663 (42.9) | 0.00 |
| **Health conditions**  Dementia  History of delirium  Depression  Incontinence  Pressure injuries  Malnutrition | 34411 (52.7)  4441 (6.80)  14168 (21.7)  21010 (32.2)  1812 (2.80)  4264 (6.50) | 840 (53.3)  144 (9.10)  324 (20.6)  414 (26.3)  56 (3.60)  125 (7.90) | 0.01  0.09  0.03  0.13  0.05  0.05 | 835 (54.0)  157 (10.2)  333 (21.6)  403 (26.1)  58 (3.80)  125 (8.10) | 828 (53.6)  142 (9.20)  318 (20.6)  408 (26.4)  55.0 (3.60)  124 (8.00) | 0.01  0.03  0.02  0.01  0.01  0.01 |
| **SEIFA RSD quintile**  1 (Most disadvantaged)  2  3  4  5 (Least disadvantaged)  Missing | 7273 (11.1)  8628 (13.2)  13082 (20.0)  13852 (21.2)  20866 (31.9)  1633 (2.50) | 232 (14.7)  244 (15.5)  247 (15.7)  363 (23.0)  399 (25.3)  90 (5.70) | 0.26 | 224 (14.5)  215 (13.9)  261 (16.9)  366 (23.7)  397 (25.7)  82 (5.30) | 222 (14.4)  266 (15.1)  245 (15.9)  358 (23.2)  398 (25.8)  89 (5.80) | 0.05 |
| **Medications (90 days)^1^ (Mean, SD)** | 7.98 (4.71) | 8.01 (4.77) | 0.01 | 8.05 (4.78) | 8.02 (4.75) | 0.01 |
| **Sedative load (90 days)^1^ (Mean, SD)** | 1.64 (1.86) | 1.96 (2.20) | 0.16 | 1.98 (2.14) | 1.95 (2.18) | 0.01 |
| **Comorbidities (Mean, SD)** | 5.25 (2.72) | 5.18 (2.86) | 0.03 | 5.20 (2.82) | 5.21 (2.83) | 0.01 |
| **Hospital encounters (365 days^1^; Mean, SD)**  Hospitalisations  Length of Stay  ED Presentations | 2.43 (7.19)  29.0 (71.4)  1.78 (2.23) | 2.86 (7.99)  72.2 (212)  2.09 (2.30) | 0.06  0.27  0.14 | 2.78 (6.55)  59.6 (182)  2.16 (2.42) | 2.86 (8.06)  65.5 (149)  2.08 (2.30) | 0.01  0.04  0.04 |
| **Primary health care (365 days^1^; Mean, SD)**  General attendances  Urgent after hour attendances  Health assessments  Chronic disease management plans  Pain medicine attendances  Geriatric medicine attendances | 14.7 (11.2)  0.40 (1.24)  0.37 (0.55)  1.36 (1.64)  0.17 (0.58)  0.16 (0.50) | 12.2 (10.0)  0.42 (0.98)  0.29 (0.50)  1.10 (1.50)  0.10 (0.56)  0.08 (0.37) | 0.23  0.02  0.15  0.16  0.12  0.18 | 12.7 (11.2)  0.45 (1.16)  0.32 (0.51)  1.15 (1.58)  0.12 (0.62)  0.09 (0.41) | 12.3 (9.99)  0.43 (0.98)  0.30 (0.50)  1.11 (1.51)  0.11 (0.57)  0.08 (0.37) | 0.03  0.02  0.01  0.03  0.03  0.01 |
| **Respite prior to LTCF entry, any (N, %)** | 31400 (48.1) | 418 (26.5) | 0.46 | 407 (26.3) | 416 (26.7) | 0.01 |
| **State of service (N, %)**  New South Wales  Queensland  Victoria | 33714 (51.6)  14872 (22.8)  16748 (25.6) | 77.0 (4.90)  234 (14.9)  1264 (80.3) | 1.44 | 67.0 (4.30)  247 (16.1)  1231 (79.7) | 75.0 (4.90)  234 (15.3)  1236 (79.9) | 0.03 |
| **Year of care entry (N, %)**  2013  2014  2015  2016  2017  2018 | 10087 (15.4)  9771 (15.0)  10438 (16.0)  11305 (17.3)  11860 (18.2)  11873 (18.2) | 217 (13.8)  219 (13.9)  286 (18.2)  280 (17.8)  286 (18.2)  287 (18.2) | 0.08 | 212 (13.7)  215 (13.9)  267 (17.3)  286 (18.5)  283 (18.3)  282 (18.3) | 215 (13.9)  216 (14.1)  277 (17.9)  270 (17.6)  282 (18.3)  285 (18.4) | 0.03 |
| Notes: 1, refers to days prior to LTCF entry; 2, A 1:1 nearest neighbour matching without replacement was used, with a caliper of 0.05.  Abbreviations: ASMD; average standardised mean difference; SD, standard deviation; SEIFA, socio-economic indexes for areas; RSD, relative socio-economic disadvantage; LTCF, long-term care facility; ED, emergency department; GP, general practitioner; NSW, New South Wales; QLD, Queensland; VIC, Victoria | | | | | | |

# Table S7. Long-term care facility residents’ characteristics, not-for-profit vs government in inner regional areas, before and after matching

|  | **Before matching** | | | **After matching^2^** | | |
| --- | --- | --- | --- | --- | --- | --- |
|  | **Not-for-profit**  **N=30882**  **n (% of N)** | **Govt**  **N=5294**  **n (% of N)** | **ASMD** | **Not-for-profit**  **N=5015**  **n (% of N)** | **Govt**  **N=5015**  **n (% of N)** | **ASMD** |
| **Male (N, %)** | 12071 (39.1) | 2237 (42.3) | 0.07 | 2092 (41.7) | 2083 (41.5) | 0.004 |
| **Age (Mean, SD)** | 84.3 (7.07) | 83.6 (7.33) | 0.09 | 83.9 (6.97) | 83.7 (7.31) | 0.022 |
| **Partner status; Yes, partner (N, %)** | 11546 (37.4) | 2086 (39.4) | 0.04 | 1861 (37.1) | 1977 (39.4) | 0.048 |
| **Health conditions**  Dementia  History of delirium  Depression  Incontinence  Pressure injuries  Malnutrition | 14402 (46.6)  1438 (4.70)  6436 (20.8)  9034 (29.3)  765 (2.50)  1617 (5.20) | 2618 (49.5)  245 (4.60)  1066 (20.1)  1475 (27.9)  135 (2.60)  365 (6.90) | 0.06  0.00  0.02  0.03  0.01  0.07 | 2442 (48.7)  209 (4.20)  970 (19.3)  1377 (27.5)  130 (2.60)  320 (6.40) | 2463 (49.1)  229 (4.60)  1015 (20.2)  1387 (27.7)  126 (2.50)  348 (6.90) | 0.008  0.02  0.023  0.004  0.005  0.022 |
| **SEIFA RSD quintile**  1 (Most disadvantaged)  2  3  4  5 (Least disadvantaged)  Missing | 9158 (29.7)  9093 (29.4)  8168 (26.4)  2169 (7.00)  1839 (6.00)  455 (1.50) | 1389 (26.2)  1760 (33.2)  1142 (21.6)  461 (8.70)  338 (6.40)  204 (3.90) | 0.21 | 1299 (25.9)  1694 (33.8)  1052 (21.0)  434 (8.70)  367 (7.30)  169 (3.40) | 1323 (26.4)  1671 (33.3)  1070 (21.3)  438 (8.70)  336 (6.70)  177 (3.50) | 0.029 |
| **Medications (90 days)^1^ (Mean, SD)** | 8.11 (4.57) | 8.13 (4.64) | 0.01 | 8.14 (4.65) | 8.12 (4.58) | 0.005 |
| **Sedative load (90 days)^1^ (Mean, SD)** | 1.77 (1.92) | 1.76 (1.95) | 0.00 | 1.79 (1.97) | 1.77 (1.95) | 0.008 |
| **Comorbidities (Mean, SD)** | 5.36 (2.69) | 5.33 (2.73) | 0.01 | 5.40 (2.73) | 5.34 (2.72) | 0.021 |
| **Hospital encounters (365 days^1^; Mean, SD)**  Hospitalisations  Length of Stay  ED Presentations | 2.30 (6.29)  24.1 (63.3)  2.04 (2.79) | 2.61 (7.73)  63.6 (187)  1.68 (2.47) | 0.04  0.28  0.14 | 2.33 (5.44)  41.4 (120)  1.59 (1.95) | 2.39 (5.91)  53.9 (153)  1.66 (2.46) | 0.01  0.091  0.033 |
| **Primary health care (365 days^1^; Mean, SD)**  General attendances  Urgent after hour attendances  Health assessments  Chronic disease management plans  Pain medicine attendances  Geriatric medicine attendances | 15.9 (12.8)  0.14 (0.65)  0.35 (0.51)  1.49 (1.77)  0.11 (0.51)  0.10 (0.37) | 15.6 (13.0)  0.28 (0.82)  0.26 (0.46)  1.16 (1.75)  0.09 (0.49)  0.07 (0.34) | 0.03  0.18  0.18  0.18  0.05  0.07 | 16.1 (14.3)  0.27 (0.95)  0.27 (0.47)  1.21 (1.68)  0.09 (0.45)  0.07 (0.34) | 15.7 (13.1)  0.26 (0.76)  0.26 (0.46)  1.19 (1.76)  0.09 (0.50)  0.07 (0.34) | 0.028  0.01  0.006  0.008  0.002  0.001 |
| **Respite prior to LTCF entry, any (N, %)** | 17136 (55.5) | 2348 (44.4) | 0.22 | 2342 (46.7) | 2279 (45.4) | 0.025 |
| **State of service (N, %)**  New South Wales  Queensland  Victoria | 15961 (51.7)  7873 (25.5)  7048 (22.8) | 525 (9.90)  578 (10.9)  4191 (79.2) | 1.39 | 400 (8.00)  629 (12.5)  3986 (79.5) | 501 (10.0)  578 (11.5)  3936 (78.5) | 0.074 |
| **Year of care entry (N, %)**  2013  2014  2015  2016  2017  2018 | 4466 (14.5)  4691 (15.2)  5094 (16.5)  5188 (16.8)  5695 (18.4)  5748 (18.6) | 789 (14.9)  900 (17.0)  958 (18.1)  866 (16.4)  893 (16.9)  888 (16.8) | 0.08 | 700 (14.0)  815 (16.3)  905 (18.0)  826 (16.5)  853 (17.0)  916 (18.3) | 742 (14.8)  842 (16.8)  897 (17.9)  823 (16.4)  855 (17.0)  856 (17.1) | 0.039 |
| Notes: 1, refers to days prior to LTCF entry; 2, A 1:1 nearest neighbour matching without replacement was used, with a caliper of 0.05.  Abbreviations: ASMD; average standardised mean difference; SD, standard deviation; SEIFA, socio-economic indexes for areas; RSD, relative socio-economic disadvantage; LTCF, long-term care facility; ED, emergency department; GP, general practitioner; NSW, New South Wales; QLD, Queensland; VIC, Victoria | | | | | | |

# Table S8. Long-term care facility residents’ characteristics, not-for-profit vs government in outer regional, remote and very remote areas, before and after matching

|  | **Before matching** | | | **After matching^2^** | | |
| --- | --- | --- | --- | --- | --- | --- |
|  | **Not-for-profit**  **N=8928**  **n (% of N)** | **Govt**  **N=2092**  **n (% of N)** | **ASMD** | **Not-for-profit**  **N=1576**  **n (% of N)** | **Govt**  **N=1576**  **n (% of N)** | **ASMD** |
| **Male (N, %)** | 3624 (40.6) | 867 (41.4) | 0.02 | 621 (39.4) | 638 (40.5) | 0.02 |
| **Age (Mean, SD)** | 83.8 (7.10) | 84.1 (7.06) | 0.05 | 83.9 (7.07) | 84.0 (7.13) | 0.02 |
| **Partner status; Yes, partner (N, %)** | 3091 (34.6) | 784 (37.5) | 0.06 | 555 (35.2) | 573 (36.4) | 0.02 |
| **Health conditions**  Dementia  History of delirium  Depression  Incontinence  Pressure injuries  Malnutrition | 4223 (47.3)  340 (3.80)  1989 (22.3)  2460 (27.6)  192 (2.20)  460 (5.20) | 973 (46.5)  80.0 (3.80)  441 (21.1)  533 (25.5)  66.0 (3.20)  121 (5.80) | 0.02  0.00  0.03  0.05  0.06  0.03 | 769 (48.8)  61.0 (3.90)  354 (22.5)  437 (27.7)  45.0 (2.90)  86.0 (5.50) | 763 (48.4)  57.0 (3.60)  333 (21.1)  435 (27.6)  40.0 (2.50)  80.0 (5.10) | 0.01  0.01  0.03  0.00  0.02  0.02 |
| **SEIFA RSD quintile**  1 (Most disadvantaged)  2  3  4  5 (Least disadvantaged)  Missing | 3126 (35.0)  3374 (37.8)  1780 (19.9)  340 (3.80)  194 (2.20)  114 (1.30) | 611 (29.2)  862 (41.2)  357 (17.1)  145 (6.90)  52.0 (2.50)  65.0 (3.10) | 0.23 | 545 (34.6)  589 (37.4)  278 (17.6)  75.0 (4.80)  41.0 (2.60)  48.0 (3.00) | 486 (30.8)  631 (40.0)  285 (18.1)  88.0 (5.60)  41.0 (2.60)  45.0 (2.90) | 0.09 |
| **Medications (90 days)^1^ (Mean, SD)** | 7.66 (4.44) | 8.01 (4.44) | 0.08 | 7.86 (4.36) | 7.83 (4.44) | 0.01 |
| **Sedative load (90 days)^1^ (Mean, SD)** | 1.67 (1.84) | 1.91 (1.97) | 0.13 | 1.78 (1.80) | 1.81 (1.92) | 0.02 |
| **Comorbidities (Mean, SD)** | 5.14 (2.67) | 5.4 (2.68) | 0.10 | 5.34 (2.69) | 5.27 (2.70) | 0.03 |
| **Health service use (365 days^1^; Mean, SD)**  Hospitalisations  Length of Stay  ED Presentations | 2.30 (5.02)  16.5 (40.2)  2.45 (3.82) | 2.60 (5.7)  45.2 (137)  1.92 (3.75) | 0.06  0.28  0.14 | 2.34 (5.1)  25.8 (71.7)  2.13 (5.15) | 2.37 (4.54)  27.9 (79.0)  2.12 (4.14) | 0.01  0.03  0.00 |
| **Health service use (365 days^1^; Mean, SD)**  General attendances  Urgent after hour attendances  Health assessments  Chronic disease management plans  Pain medicine attendances  Geriatric medicine attendances | 15.7 (13.2)  0.17 (0.71)  0.32 (0.50)  1.28 (1.76)  0.08 (0.38)  0.06 (0.29) | 17.6 (14.03)  0.28 (1.12)  0.29 (0.49)  0.97 (1.45)  0.05 (0.37)  0.04 (0.24) | 0.14  0.12  0.06  0.19  0.06  0.09 | 16.2 (13.3)  0.17 (0.63)  0.31 (0.49)  1.09 (1.60)  0.05 (0.35)  0.04 (0.25) | 16.1 (12.0)  0.19 (0.75)  0.30 (0.49)  1.00 (1.46)  0.06 (0.37)  0.04 (0.26) | 0.00  0.03  0.01  0.06  0.02  <0.001 |
| **Respite prior to LTCF entry, any (N, %)** | 5094 (57.1) | 1296 (62.0) | 0.10 | 937 (59.5) | 966 (61.3) | 0.04 |
| **State of service (N, %)**  New South Wales  Queensland  Victoria | 3945 (44.2)  4057 (45.4)  926 (10.4) | 237 (11.3)  492 (23.5)  1363 (65.2) | 1.41 | 226 (14.3)  490 (31.1)  860 (54.6) | 224 (14.2)  492 (31.2)  860 (54.6) | 0.00 |
| **Year of care entry (N, %)**  2013  2014  2015  2016  2017  2018 | 1366 (15.3)  1341 (15.0)  1401 (15.7)  1478 (16.6)  1677 (18.8)  1665 (18.6) | 318 (15.2)  327 (15.6)  382 (18.3)  320 (15.3)  345 (16.5)  400 (19.1) | 0.09 | 227 (14.4)  233 (14.8)  279 (17.7)  233 (14.8)  283 (18.0)  321 (20.4) | 225 (14.3)  245 (15.5)  274 (17.4)  241 (15.3)  274 (17.4)  317 (20.1) | 0.03 |
| Notes: 1, refers to days prior to LTCF entry; 2, A 1:1 nearest neighbour matching without replacement was used, with a caliper of 0.05.  Abbreviations: ASMD; average standardised mean difference; SD, standard deviation; SEIFA, socio-economic indexes for areas; RSD, relative socio-economic disadvantage; LTCF, long-term care facility; ED, emergency department; GP, general practitioner; NSW, New South Wales; QLD, Queensland; VIC, Victoria | | | | | | |

# Table S9. Long-term care facility residents’ characteristics, for-profit vs government in metropolitan areas, before and after matching

|  | **Before matching** | | | **After matching^2^** | | |
| --- | --- | --- | --- | --- | --- | --- |
|  | **For-profit**  **N=74600**  **n (% of N)** | **Govt**  **N=1575**  **n (% of N)** | **ASMD** | **For-profit**  **N=1548**  **n (% of N)** | **Govt**  **N=1548**  **n (% of N)** | **ASMD** |
| **Male (N, %)** | 29481 (39.5) | 808 (51.3) | 0.24 | 766 (49.5) | 785 (50.7) | 0.03 |
| **Age (Mean, SD)** | 84.2 (7.23) | 81.6 (8.08) | 0.34 | 81.9 (7.43) | 81.8 (8.02) | 0.01 |
| **Partner status; Yes, partner (N, %)** | 28230 (37.8) | 675 (42.9) | 0.10 | 658 (42.5) | 664 (42.9) | 0.01 |
| **Health conditions**  Dementia  History of delirium  Depression  Incontinence  Pressure injuries  Malnutrition | 39798 (53.3)  5819 (7.80)  15855 (21.3)  25612 (34.3)  1959 (2.60)  5105 (6.80) | 840 (53.3)  144 (9.10)  324 (20.6)  414 (26.3)  56.0 (3.60)  125 (7.90) | <0.001  0.05  0.02  0.18  0.05  0.04 | 842 (54.4)  134 (8.70)  321 (20.7)  405 (26.2)  45.0 (2.90)  145 (9.40) | 831 (53.7)  140 (9.30)  318 (20.5)  412 (26.6)  55.0 (3.60)  123 (8.00) | 0.01  0.01  0.01  0.01  0.04  0.05 |
| **SEIFA RSD quintile**  1 (Most disadvantaged)  2  3  4  5 (Least disadvantaged)  Missing | 9511 (12.7)  9560 (12.8)  12677 (17.0)  17677 (23.7)  23243 (31.2)  1932 (2.60) | 232 (14.7)  244 (15.5)  247 (15.7)  363 (23.0)  399 (25.3)  90.0 (5.70) | 0.21 | 216 (14.0)  231 (14.9)  256 (16.5)  337 (21.8)  418 (27.0)  90.0 (5.80) | 226 (14.6)  239 (15.4)  243 (15.7)  361 (23.3)  396 (25.6)  83.0 (5.40) | 0.06 |
| **Medications (90 days)^1^ (Mean, SD)** | 8.18 (4.80) | 8.01 (4.77) | 0.04 | 7.96 (4.89) | 8.05 (4.76) | 0.02 |
| **Sedative load (90 days)^1^ (Mean, SD)** | 1.78 (1.95) | 1.96 (2.20) | 0.09 | 1.95 (2.11) | 1.96 (2.20) | 0.01 |
| **Comorbidities (Mean, SD)** | 5.37 (2.73) | 5.18 (2.86) | 0.07 | 5.18 (2.85) | 5.21 (2.84) | 0.01 |
| **Health service use (365 days^1^; Mean, SD)**  Hospitalisations  Length of Stay  ED Presentations | 2.66 (7.80)  30.8 (57.7)  1.81 (2.25) | 2.86 (7.99)  72.2 (212)  2.09 (2.30) | 0.03  0.27  0.12 | 2.68 (6.55)  51.8 (143)  1.99 (2.36) | 2.85 (8.05)  63.8 (141)  2.06 (2.28) | 0.02  0.08  0.03 |
| **Health service use (365 days^1^; Mean, SD)**  General attendances  Urgent after hour attendances  Health assessments  Chronic disease management plans  Pain medicine attendances  Geriatric medicine attendances | 14.7 (10.9)  0.52 (1.46)  0.38 (0.56)  1.34 (1.65)  0.15 (0.53)  0.14 (0.47) | 12.2 (9.97)  0.42 (0.98)  0.29 (0.50)  1.10 (1.50)  0.1 (0.56)  0.08 (0.37) | 0.24  0.08  0.16  0.15  0.08  0.13 | 12.2 (10.4)  0.36 (0.78)  0.30 (0.51)  0.19 (1.67)  0.12 (0.51)  0.11 (0.40) | 12.3 (9.99)  0.43 (0.98)  0.30 (0.50)  1.11 (0.57)  0.11 (0.57)  0.08 (0.37) | 0.01  0.08  0.00  0.03  0.05  0.03 |
| **Respite prior to LTCF entry, any (N, %)** | 37274 (50.0) | 418 (26.5) | 0.50 | 417 (26.9) | 417 (26.9) | <0.001 |
| **State of service (N, %)**  New South Wales  Queensland  Victoria | 26761 (35.9)  16465 (22.1)  31374 (42.1) | 77.0 (4.90)  234 (14.9)  1264 (80.3) | 0.95 | 52.0 (3.40)  252 (16.3)  1244 (80.4) | 73.0 (4.70)  234 (15.1)  1241 (80.2) | 0.07 |
| **Year of care entry (N, %)**  2013  2014  2015  2016  2017  2018 | 10166 (13.6)  11816 (15.8)  12420 (16.6)  12432 (16.7)  13667 (18.3)  14099 (18.9) | 217 (13.8)  219 (13.9)  286 (18.2)  280 (17.8)  286 (18.2)  287 (18.2) | 0.07 | 219 (14.1)  226 (14.6)  279 (18.0)  274 (17.7)  290 (18.7)  260 (16.8) | 213 (13.8)  216 (14.3)  283 (18.3)  271 (17.5)  283 (18.3)  282 (18.2) | 0.04 |
| Notes: 1, refers to days prior to LTCF entry; 2, A 1:1 nearest neighbour matching without replacement was used, with a caliper of 0.05.  Abbreviations: ASMD; average standardised mean difference; SD, standard deviation; SEIFA, socio-economic indexes for areas; RSD, relative socio-economic disadvantage; LTCF, long-term care facility; ED, emergency department; GP, general practitioner; NSW, New South Wales; QLD, Queensland; VIC, Victoria | | | | | | |

# Table S10. Long-term care facility residents’ characteristics, for-profit vs government in inner regional areas, before and after matching

|  | **Before matching** | | | **After matching^2^** | | |
| --- | --- | --- | --- | --- | --- | --- |
|  | **For-profit**  **N=13226**  **n (% of N)** | **Govt**  **N=5294**  **n (% of N)** | **ASMD** | **For-profit**  **N=4625**  **n (% of N)** | **Govt**  **N=4625**  **n (% of N)** | **ASMD** |
| **Male (N, %)** | 5592 (42.3) | 2237 (42.3) | 0.00 | 1933 (41.8) | 1943 (42.0) | 0.00 |
| **Age (Mean, SD)** | 83.7 (7.25) | 83.6 (7.33) | 0.01 | 83.61 (7.30) | 83.57 (7.34) | 0.01 |
| **Partner status; Yes, partner (N, %)** | 5366 (40.6) | 2086 (39.4) | 0.02 | 1793 (38.8) | 1826 (39.5) | 0.02 |
| **Health conditions**  Dementia  History of delirium  Depression  Incontinence  Pressure injuries  Malnutrition | 6634 (50.2)  778 (5.90)  2774 (21.0)  4435 (33.5)  395 (3.00)  784 (5.90) | 2618 (49.5)  245 (4.60)  1066 (20.1)  1475 (27.9)  135 (2.60)  365 (6.90) | 0.01  0.06  0.02  0.12  0.03  0.04 | 2273 (49.1)  224 (4.80)  952 (20.6)  1315 (28.4)  121 (2.60)  303 (6.60) | 2293 (49.6)  217 (4.70)  943 (20.4)  1339 (29.0)  121 (2.60)  318 (6.90) | 0.01  0.01  0.01  0.01  <0.001  0.01 |
| **SEIFA IRSD quintile**  1 (Most disadvantaged)  2  3  4  5 (Least disadvantaged)  Missing | 3361 (25.4)  3245 (24.5)  3243 (24.5)  1506 (11.4)  1510 (11.4)  361 (2.70) | 1389 (26.2)  1760 (33.2)  1142 (21.6)  461 (8.70)  338 (6.40)  204 (3.90) | 0.27 | 1111 (24.0)  1443 (31.2)  1065 (23.0)  475 (10.3)  336 (7.30)  195 (4.20) | 1100 (23.8)  1485 (32.1)  1061 (22.9)  451 (9.80)  336 (7.30)  192 (4.20) | 0.02 |
| **Medications (90 days)^1^ (Mean, SD)** | 8.09 (4.62) | 8.13 (4.64) | 0.01 | 8.22 (4.64) | 8.14 (4.63) | 0.02 |
| **Sedative load (90 days)^1^ (Mean, SD)** | 1.83 (1.94) | 1.76 (1.95) | 0.03 | 1.82 (1.98) | 1.78 (1.95) | 0.02 |
| **Comorbidities (Mean, SD)** | 5.32 (2.70) | 5.33 (2.73) | 0.00 | 5.35 (2.71) | 5.32 (2.71) | 0.01 |
| **Hospital encounters (365 days^1^; Mean, SD)**  Hospitalisations  Length of Stay  ED Presentations | 2.31 (6.09)  27.2 (54.5)  1.97 (2.35) | 2.61 (7.73)  63.6 (187)  1.68 (2.47) | 0.04  0.27  0.12 | 2.37 (5.73)  38.9 (79.7)  1.67 (2.02) | 2.34 (5.87)  46.3 (121)  1.72 (2.56) | 0.01  0.07  0.02 |
| **Primary health care (365 days^1^; Mean, SD)**  General attendances  Urgent after hour attendances  Health assessments  Chronic disease management plans  Pain medicine attendances  Geriatric medicine attendances | 15.5 (12.2)  0.15 (0.61)  0.33 (0.51)  1.36 (1.70)  0.10 (0.45)  0.09 (0.35) | 15.6 (13.0)  0.28 (0.82)  0.26 (0.46)  1.16 (1.75)  0.09 (0.49)  0.07 (0.34) | 0.00  0.18  0.15  0.12  0.03  0.04 | 15.2 (12.0)  0.22 (0.69)  0.26 (0.47)  1.19 (1.66)  0.08 (0.39)  0.06 (0.32) | 15.4 (12.7)  0.23 (0.68)  0.27 (0.46)  1.21 (1.79)  0.09 (0.51)  0.07 (0.35) | 0.02  0.01  0.01  0.02  0.03  0.03 |
| **Respite prior to LTCF entry, any (N, %)** | 7525 (56.9) | 2348 (44.4) | 0.25 | 2136 (46.2) | 2130 (46.1) | 0.00 |
| **State of service (N, %)**  New South Wales  Queensland  Victoria | 4976 (37.6)  2248 (17.0)  6002 (45.4) | 525 (9.90)  578 (10.9)  4191 (79.2) | 0.78 | 424 (9.20)  503 (10.9)  3698 (80.0) | 463 (10.0)  514 (11.1)  3648 (78.9) | 0.03 |
| **Year of care entry (N, %)**  2013  2014  2015  2016  2017  2018 | 1812 (13.7)  1927 (14.6)  2423 (18.3)  2378 (18.0)  2273 (17.2)  2413 (18.2) | 789 (14.9)  900 (17.0)  958 (18.1)  866 (16.4)  893 (16.9)  888 (16.8) | 0.09 | 658 (14.2)  750 (16.2)  860 (18.6)  774 (16.7)  805 (17.4)  778 (16.8) | 673 (14.6)  749 (16.2)  857 (18.5)  774 (16.7)  784 (17.0)  788 (17.0) | 0.02 |
| Notes: 1, refers to days prior to LTCF entry; 2, A 1:1 nearest neighbour matching without replacement was used, with a caliper of 0.05.  Abbreviations: ASMD; average standardised mean difference; SD, standard deviation; SEIFA, socio-economic indexes for areas; RSD, relative socio-economic disadvantage; LTCF, long-term care facility; ED, emergency department; GP, general practitioner; NSW, New South Wales; QLD, Queensland; VIC, Victoria | | | | | | |

# Table S11. Long-term care facility residents’ characteristics, for-profit vs government in outer regional, remote and very remote areas, before and after matching

|  | **Before matching** | | | **After matching^2^** | | |
| --- | --- | --- | --- | --- | --- | --- |
|  | **For-profit**  **N=3148**  **n (% of N)** | **Govt**  **N=2092**  **n (% of N)** | **ASMD** | **For-profit**  **N=1588**  **n (% of N)** | **Govt**  **N=1588**  **n (% of N)** | **ASMD** |
| **Male (N, %)** | 1366 (43.4) | 867 (41.4) | 0.04 | 640 (40.3) | 659 (41.5) | 0.02 |
| **Age (Mean, SD)** | 83.6 (7.35) | 84.1 (7.06) | 0.07 | 83.7 (7.27) | 83.8 (7.22) | 0.01 |
| **Partner status; Yes, partner (N, %)** | 1257 (39.9) | 784 (37.5) | 0.05 | 582 (36.6) | 584 (36.8) | 0.00 |
| **Health conditions**  Dementia  History of delirium  Depression  Incontinence  Pressure injuries  Malnutrition | 1542 (49.0)  165 (5.20)  724 (23.0)  1130 (35.9)  80.0 (2.50)  203 (6.40) | 973 (46.5)  80.0 (3.80)  441 (21.1)  533 (25.5)  66.0 (3.20)  121 (5.80) | 0.05  0.07  0.05  0.23  0.04  0.03 | 770 (48.5)  71.0 (4.50)  357 (22.5)  430 (27.1)  42.0 (2.60)  102 (6.40) | 762 (48.0)  66.0 (4.20)  338 (21.3)  435 (27.4)  51.0 (3.20)  98.0 (6.20) | 0.01  0.02  0.03  0.01  0.03  0.01 |
| **SEIFA IRSD quintile**  1 (Most disadvantaged)  2  3  4  5 (Least disadvantaged)  Missing | 788 (25.0)  1590 (50.5)  426 (13.5)  182 (5.80)  83.0 (2.60)  79.0 (2.50) | 611 (29.2)  862 (41.2)  357 (17.1)  145 (6.90)  52.0 (2.50)  65.0 (3.10) | 0.19 | 514 (32.4)  622 (39.2)  282 (17.8)  92.0 (5.80)  41.0 (2.60)  37.0 (2.30) | 463 (29.2)  662 (41.7)  283 (17.8)  98.0 (6.20)  42.0 (2.60)  40.0 (2.50) | 0.07 |
| **Medications (90 days)^1^ (Mean, SD)** | 7.77 (4.45) | 8.01 (4.44) | 0.05 | 7.67 (4.50) | 7.79 (4.42) | 0.03 |
| **Sedative load (90 days)^1^ (Mean, SD)** | 1.80 (1.90) | 1.91 (1.97) | 0.06 | 1.79 (1.92) | 1.82 (1.93) | 0.01 |
| **Comorbidities (Mean, SD)** | 5.23 (2.70) | 5.40 (2.68) | 0.06 | 5.24 (2.73) | 5.27 (2.68) | 0.01 |
| **Hospital encounters (365 days^1^; Mean, SD)**  Hospitalisations  Length of Stay  ED Presentations | 2.12 (4.58)  18.6 (49.3)  2.24 (2.72) | 2.59 (5.70)  45.2 (136)  1.92 (3.75) | 0.09  0.26  0.10 | 2.33 (6.10)  25.0 (64.7)  2.02 (2.39) | 2.18 (2.90)  27.1 (87.5)  2.22 (3.86) | 0.03  0.03  0.06 |
| **Primary health care (Mean, SD)**  General attendances  Urgent after hour attendances  Health assessments  Chronic disease management plans  Pain medicine attendances  Geriatric medicine attendances | 14.5 (11.7)  0.19 (0.69)  0.33 (0.52)  1.15 (1.66)  0.08 (0.40)  0.07 (0.31) | 17.6 (14.0)  0.28 (1.12)  0.29 (0.49)  0.97 (1.45)  0.05 (0.37)  0.04 (0.24) | 0.24  0.10  0.07  0.12  0.07  0.11 | 14.7 (11.8)  0.13 (0.54)  0.28 (0.49)  1.00 (1.63)  0.04 (0.40)  0.03 (0.22) | 14.9 (10.7)  0.11 (0.55)  0.29 (0.49)  0.99 (1.47)  0.05 (0.35)  0.04 (0.25) | 0.02  0.02  0.02  0.01  0.02  0.02 |
| **Respite prior to LTCF entry, any (N, %)** | 2092 (66.5) | 1296 (62.0) | 0.09 | 1002 (63.1) | 999 (62.9) | 0.00 |
| **State of service (N, %)**  New South Wales  Queensland  Victoria | 744 (23.6)  1195 (38.0)  1209 (38.4) | 237 (11.3)  492 (23.5)  1363 (65.2) | 0.56 | 217 (13.7)  482 (30.4)  889 (56.0) | 210 (13.2)  488 (30.7)  890 (56.0) | 0.01 |
| **Year of care entry (N, %)**  2013  2014  2015  2016  2017  2018 | 429 (13.6)  471 (15.0)  554 (17.6)  595 (18.9)  548 (17.4)  551 (17.5) | 318 (15.2)  327 (15.6)  382 (18.3)  320 (15.3)  345 (16.5)  400 (19.1) | 0.11 | 233 (14.7)  229 (14.4)  283 (17.8)  270 (17.0)  262 (16.5)  311 (19.6) | 232 (14.6)  247 (15.6)  290 (18.3)  250 (15.7)  264 (16.6)  305 (19.2) | 0.05 |
| Notes: 1, refers to days prior to LTCF entry; 2, A 1:1 nearest neighbour matching without replacement was used, with a caliper of 0.05.  Abbreviations: ASMD; average standardised mean difference; SD, standard deviation; SEIFA, socio-economic indexes for areas; RSD, relative socio-economic disadvantage; LTCF, long-term care facility; ED, emergency department; GP, general practitioner; NSW, New South Wales; QLD, Queensland; VIC, Victoria | | | | | | |

# Table S12. Descriptive outcomes (follow up, mortality, emergency department presentations, unplanned hospitalisations, and days in hospital) in the first 12 months of long-term care facility entry for matched facility ownership cohorts, by geographical remoteness.

|  | **Metropolitan** | | **Inner regional** | | **Outer regional, remote and very remote** | |
| --- | --- | --- | --- | --- | --- | --- |
| **Not-for-profit vs For-profit** | **Not-for-profit**  **(n=57889)** | **For-profit**  **(n=57889)** | **Not-for-profit**  **(n=12306)** | **For-profit**  **(n=12306)** | **Not-for-profit**  **(n=2752)** | **For-profit**  **(n=2752)** |
| **Follow up days (365 days), median (IQR)^1^** | 311 (310-312) | 302 (301-303) | 309 (307-311) | 298 (295-300) | 309 (305-313) | 308 (304-312) |
| **Mortality (cumulative incidence %, 95% CI)^2^** | 26.5 (26.1-26.8) | 29.3 (29.0-29.7) | 27.8 (27.0-28.5) | 31.3 (30.4-32.1) | 27.4 (25.7-29.1) | 27.1 (25.4-28.7) |
| **ED presentations**  Cumulative incidence (%, 95% CI)^2^  Rate (per 100 resident days; mean, 95%CI)^3^ | 49.7 (49.3-50.1)  51.4 (50.2-52.6) | 50.5 (50.1-50.9)  57.8 (56.4-59.2) | 44.7 (43.8-45.5)  43.3 (41.4-45.3) | 45.9 (45.0-46.8)  51.1 (48.3-53.8) | 47.2 (45.3-49.1)  50.0 (44.6-55.4) | 48.9 (47.0-50.7)  53.7 (47.6-59.9) |
| **Unplanned hospitalisations**  Cumulative incidence (%, 95% CI)^2^  Rate (per 100 resident days; mean, 95%CI)^3^  Days in hospital, if yes (median, IQR)^4^ | 46.2 (45.8-46.6)  45.4 (44.3-46.6)  4.20 (0.78-10.1) | 47.7 (47.3-48.1)  51.1 (49.8-52.4)  4.10 (0.79-9.74) | 36.5 (35.7-37.4)  32.3 (30.6-34.0)  3.90 (1.00-8.90) | 37.0 (36.2-37.9)  38.3 (35.9-40.7)  3.80 (0.90-8.60) | 41.4 (39.6-43.3)  41.0 (35.8-46.1)  3.70 (0.90-9.30) | 41.2 (39.4-43.1)  44.3 (38.2-50.5)  3.50 (0.70-7.90) |
| **Government vs Not-for-profit** | **Government**  **(n=1545)** | **Not-for-profit**  **(n=1545)** | **Government**  **(n=5015)** | **Not-for-profit**  **(n=5015)** | **Government**  **(n=1576)** | **Not-for-profit**  **(n=1576)** |
| **Follow up days (365 days), median (IQR)^1^** | 304 (298-309) | 306 (300-311) | 299 (296-302) | 309 (306-312) | 307 (302-313) | 314 (309-319) |
| **Mortality (cumulative incidence %, 95% CI)^2^** | 31.5 (29.2-33.8) | 28.6 (26.4-30.9) | 32.5 (31.2-33.8) | 27.9 (26.6-29.1) | 28.6 (26.3-30.8) | 25.6 (23.4-27.8) |
| **ED presentations**  Cumulative incidence (%, 95% CI)^2^  Rate (per 100 resident days; mean, 95%CI) ^3^ | 46.4 (43.9-48.4)  42.8 (38.2-47.4) | 50.9 (48.4-53.4)  51.7 (45.2-58.3) | 29.7 (28.4-31.0)  23.0 (21.3-24.7) | 40.5 (39.1-41.9)  37.6 (34.8-40.3) | 38.5 (36.0-40.9)  50.6 (36.5-64.6) | 43.5 (41.1-46.0)  43.2 (38.0-48.2) |
| **Unplanned hospitalisations**  Cumulative incidence (%, 95% CI)^2^  Rate (per 100 resident days; mean, 95%CI)^3^  Days in hospital, if yes (median, IQR)^4^ | 39.5 (37.0-41.9)  33.7 (29.5-37.9)  3.56 (0.60-8.90) | 47.2 (44.8-49.7)  45.9 (39.5-52.3)  3.00 (0.24-7.92) | 26.2 (25.0-27.4)  18.9 (17.4-20.4)  3.98 (1.30-8.60) | 35.9 (34.5-37.2)  32.2 (29.5-34.8)  3.92 (1.06-8.79) | 34.9 (32.5-37.3)  36.4 (31.2-41.6)  3.68 (1.31-8.86) | 40.3 (37.9-42.7)  36.5 (32.1-40.8)  4.37 (1.36-10.0) |
| **Government vs For-profit** | **Government**  **(n=1548)** | **For-profit**  **(n=1548)** | **Government**  **(n=4625)** | **For-profit**  **(n=4625)** | **Government**  **(n=1588)** | **For-profit**  **(n=1588)** |
| **Follow up days, median (IQR)^1^** | 304 (299-309) | 300 (294-306) | 299 (296-302) | 299 (296-302) | 305 (299-310) | 304 (299-310) |
| **Mortality (cumulative incidence %, 95% CI)^2^** | 31.3 (29.0-33.6) | 30.2 (27.9-32.5) | 32.6 (31.3-34.0) | 30.7 (29.3-32.0) | 29.3 (27.0-31.5) | 28.1 (25.9-30.3) |
| **ED presentations**  Cumulative incidence (%, 95% CI)^2^  Rate (per 100 resident days; mean, 95%CI)^3^ | 46.1 (43.6-48.5)  42.4 (37.8-47.0) | 50.6 (48.1-53.1)  58.1 (44.5-71.7) | 30.0 (28.6-31.3)  23.4 (21.6-25.2) | 44.0 (42.5-45.4)  46.4 (42.3-50.4) | 37.9 (35.5-40.3)  49.7 (35.8-63.5) | 45.0 (42.6-47.5)  49.8 (42.4-57.2) |
| **Unplanned hospitalisations**  Cumulative incidence (%, 95% CI)^2^  Rate (per 100 resident days; mean, 95% CI)^3^  Days in hospital, if yes (median, IQR)^4^ | 39.1 (36.7-41.6)  33.4 (29.2-37.7)  3.56 (0.60-8.96) | 47.5 (45.1-50.0)  52.8 (39.3-66.4)  3.29 (0.40-9.25) | 26.3 (25.0-27.6)  18.9 (17.3-20.5)  4.10 (1.30-8.60) | 37.4 (36.0-38.8)  33.7 (32.8-41.0)  3.80 (0.98-8.37) | 33.5 (31.2-35.8)  34.7 (29.6-39.8)  3.83 (1.35-9.00) | 38.3 (35.9-40.7)  41.4 (34.9-47.9)  3.67 (0.71-8.42) |
| Notes: 1, median follow up time in days within the 12-month period prior after LTCF entry; 2, cumulative incidences presented as % (95%CI); 3, rate per 100 resident-days of follow-up presented as mean (95%CI); 4, Hospital days due to unplanned hospitalisations, if any, presented as median (IQR).  Abbreviations: LTCF, long-term care facility; ED, emergency department; IQR, interquartile range CI, confidence interval; SD standard deviation. | | | | | | |
